# Supplementary material for: Repeat length as a key determinant for disease severity and antisense oligonucleotide activity in myotonic dystrophy type 1
Source: Mol Ther Methods Clin Dev. 2025 Jun 2;33(3):101502. doi: 10.1016/j.omtm.2025.101502 (PMC12221585; doi:10.1016/j.omtm.2025.101502)
Supplement: Document S1. Figure S1 and Table S1–S4 [file mmc1.pdf]

OMTM, Volume *33*

Supplemental information

Repeat length as a key determinant for disease severity and antisense oligonucleotide activity in myotonic dystrophy type 1

Najoua El Boujnouni, Lise Ripken, Marieke Willemse, Bart van der Sanden, Kornelia Neveling, Alexander Hoischen, Roland Brock, and Derick G. Wansink

Supplemental Information

Supplemental Tables and Figures

Table S1: Sequences and modifications of ASOs.[31] Underlined, 2'-O-Me; bold and non-underlined, DNA; *, phosphorothioate (PS) linkage.

Name	Sequence
**Repeat-targeting blocker**	C*A*G*C*A*G*C*A*G*C*A*G*C*A*G
**Control blocker**	G*A*C*G*A*C*G*A*C*G*A*C*G*A*C
***DMPK*-targeting gapmer**	C*G*G*A*G***C*G*G*T*T*G*T*G*A*A***C*U*G*G*C
**Control gapmer**	G*A*C*G***A*C*G*A*C*G*A*C*G*A***C*G*A*C
**Cy5-labelled (repeat-targeting blocker) ASO**	5' Cy5-C*A*G*C*A*G*C*A*G*C*A*G*C*A*G

Table S2: PCR primers used in this study.

Gene	Forward primer (5' → 3')	Reverse primer (5' → 3')	Assay
DMPK e1-2	ACTGGCCCAGGACAAGTACG	CCTCCTTAAGCCTCACCACG	RT-qPCR
DMPK e15 3'	TGCCTGCTTACTCGGGAAATT	GAGCAGCGCAAGTGAGGAG	RT-qPCR
GAPDH	CCCGCTTCGCTCTCTGCTCC	CCTTCCCCATGGTGTCTGAGCG	RT-qPCR
HPRT1	TGACACTGGCAAAACAATGCA	GGTCCTTTTCACCAGCAAGCT	RT-qPCR
MBNL1	AGGGAGATGCTCTCGGGAAAAGTG	GTTGGCTAGAGCCTGTTGGTATTGGAAAATAC	RT-PCR (splicing)
NFIX	GATGGAGAGCCCTGTTGATGACG	GTGGTGGTGGTAGCGGATGGTC	RT-PCR (splicing)
CLASP1	CAAAGTCTCCTCATCTTCGGGCACG	GCTGGGACTGTGAAACCACTTTAGC	RT-PCR (splicing)

Table S3: $(CTG)_n$ repeat length in isogenic Cas9n-treated and control cell lines determined by OGM.

Cell line	Number of triplets
**iDM2900**	2775 (p17)/2955 (p26)
**iDM2200**	2237
**iDM1200**	1204
**iDM3400**	3418
**iDM2700**	2733

Table S4: Common (CNG)$_n$ microsatellites examined in this study.

Gene	Sequence in iDM2900	Forward primer (5' → 3')	Reverse primer (5' → 3')
AR	$(CAG)_{22/23}$ CAA GAG ACT AGC CCC AGG $(CAG)_6$	TAGGGCTGGGAAGGGTCTAC	CTCTGGGACGCAACCTCTCT
ATN1	CAG CAA CAG CAA $(CAG)_8$	ACTCAGCCTTCTCTCCCATC	TGTAGGACACCTGGCTGTGA
ATXN1	$(CAG)_{12}$ CAT CAG CAT $(CAG)_{14/15}$	TTCCAGTTCATTGGGTCCTC	GTGTGTGGGATCATCGTCTG
DMPK	$(CTG)_{13}$	GAAGGGTCCTTGTAGCCGGGAA	GGAGGATGGAACACGGACGG
PPP2R2B	$(CAG)_{10}$	GCAGCAAAGAGCAGCCGCAG	CTGGTCCCACGGGAGGGCGG
TBP	$(CAG)_3$ $(CAA)_3$ $(CAG)_9$ CAA CAG CAA $(CAG)_{16/17}$ CAA CAG	TTCTCCTTGCTTTCCACAGG	GGGGAGGGATACAGTGGAGT
TCF4	$(CTG)_{11}$	CAGCACAAAGCGGAACTTGAC	GGCCATAAACGTGGCAATGTC

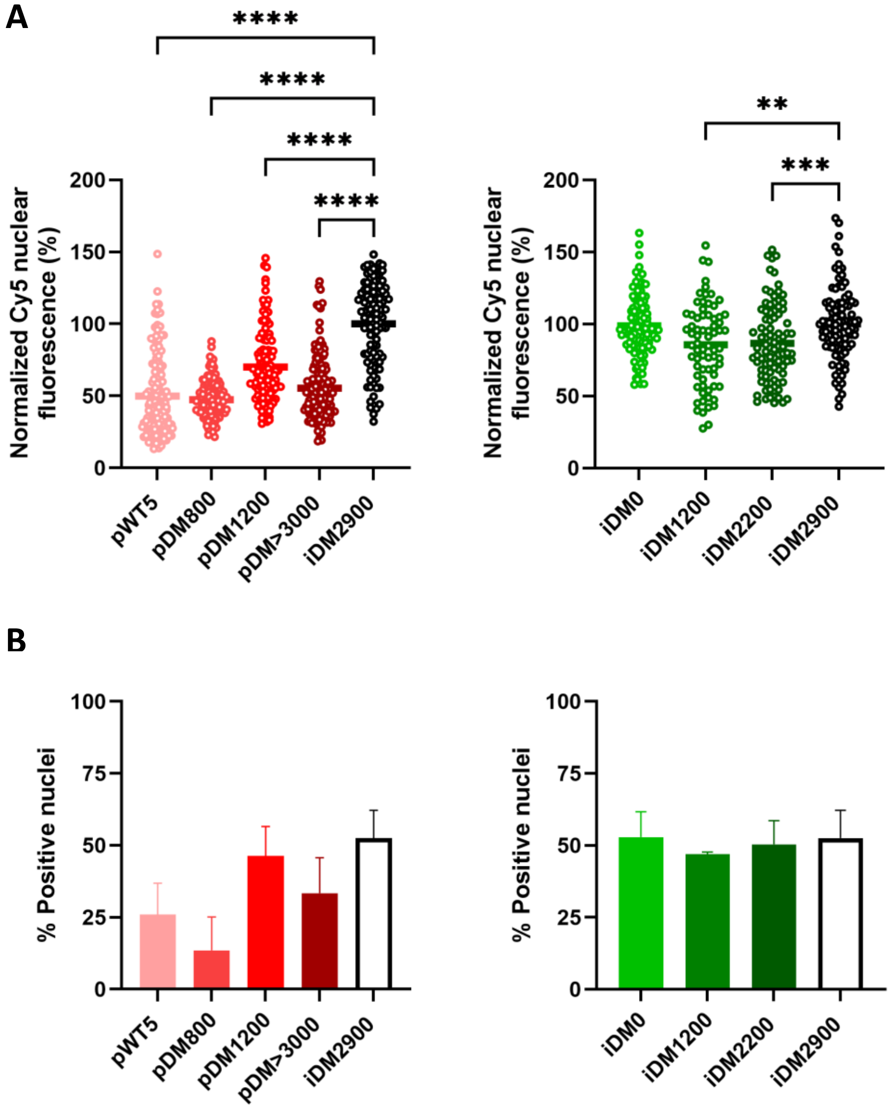

Figure S1: Pepfect14-mediated nuclear uptake of Cy5-labelled ASOs. (A) Mean nuclear (Cy5) fluorescence intensity after treatment with PepFect14/ASO nanoparticles (containing 200 nM Cy5-labelled repeat-targeting blocker ASO) for 24 hours,

together with individual data points (representing imaged nuclei) for the primary (left) and isogenic (right) cell panel. (B) The percentage of transfected ('positive') nuclei for the primary (left) and isogenic (right) cell panel. Data are presented as mean ± SD of a representative experiment (A) or mean ± SEM of three independent experiments (B). A Kruskal-Wallis (nonparametric) test with Dunn post hoc test (A and B (left)) or a one-way ANOVA with Tukey post hoc test (B, right) was used for comparison of nuclear uptake between all cell cultures. **$p < 0.01$. ***$p < 0.001$, ****$p < 0.0001$.
