# Supplementary material for: Repeat length as a key determinant for disease severity and antisense oligonucleotide activity in myotonic dystrophy type 1
Source: Mol Ther Methods Clin Dev. 2025 Jun 2;33(3):101502. doi: 10.1016/j.omtm.2025.101502 (PMC12221585; doi:10.1016/j.omtm.2025.101502)
Supplement: Document S2. Article plus supplemental information [file mmc2.pdf]

Molecular Therapy
Methods & Clinical Development
Original Article

Repeat length as a key determinant for disease severity and antisense oligonucleotide activity in myotonic dystrophy type 1

Najoua El Boujnouni,[1,5] Lise Ripken,[1,5] Marieke Willemse,[1] Bart van der Sanden,[2] Kornelia Neveling,[2] Alexander Hoischen,[2,3] Roland Brock,[1,4,6] and Derick G. Wansink[1,6]

[1]Department of Medical BioSciences, Radboud University Medical Center, Nijmegen 6525 GA, the Netherlands; [2]Department of Human Genetics, Radboud University Medical Center, Nijmegen 6525 GA, the Netherlands; [3]Department of Internal Medicine, Radboud Expertise Center for Immunodeficiency and Autoinflammation and Radboud Center for Infectious Disease, Radboud University Medical Center, Nijmegen 6525 GA, the Netherlands; [4]Department of Medical Biochemistry, College of Medicine and Medical Sciences, Arabian Gulf University, Manama, Bahrain

Myotonic dystrophy type 1 (DM1) is caused by a $(CTG)_n$ expansion in the *DMPK* gene, leading to a multisystemic manifestation and broad disease presentation. Although the DM1 phenotype and onset correlate with expansion length, understanding DM1 etiology and developing effective therapies remains challenging. Here, we investigated the contribution of repeat length on aberrant splicing and response to antisense oligonucleotides (ASOs). In primary DM1 myoblasts bearing repeat lengths of 800, 1,200, or >3,000, *DMPK* downregulation was achieved by blocking and gapmer ASOs, though splicing correction was inefficient. Further analyses revealed profound differences in *DMPK* mRNA levels. To exclude such confounding effects, we generated an isogenic myoblast panel with repeats from 0 to 2,900 triplets using a repeat-targeted CRISPR/Cas9 nickase approach. This panel revealed repeat-length dependency of aberrant splicing and nuclear MBNL1 abundance. While the blocker ASO marginally induced *DMPK* downregulation with longer repeats, its effect on splicing correction was evident, though decreased as repeat length increased. The gapmer ASO led to substantial downregulation and essentially normalized splicing levels throughout. Our study demonstrates that repeat length is central to therapeutic effectiveness, but this correlation may be obscured by genetic background, underscoring the need to consider genotypic heterogeneity in DM1 clinical trials.

INTRODUCTION

Myotonic dystrophy type 1 (DM1), the most common adult-onset neuromuscular disease,[1] is caused by a microsatellite $(CTG)_n$ repeat expansion in the 3′ untranslated region (UTR) of the dystrophia myotonica protein kinase (*DMPK*) gene.[2] Upon transcription, *DMPK* RNAs containing expanded $(CUG)_n$ sequences are retained in the nucleus and primarily sequester the splicing factor muscleblind-like splicing regulator 1 (MBNL1). Capture of the MBNL1 protein prevents the transcriptomic transition from a fetal to adult splicing pattern and as a result, several transcripts, such as the ones for chloride channel 1 (*CLCN1*), bridging integrator 1 (*BIN1*), nuclear factor IX (*NFIX*), and *MBNL1* itself, are aberrantly spliced.[3–7]

DM1-related splicing defects are causative of multisystemic effects including myotonia, muscle weakness and wasting, respiratory failure, cardiac arrythmias, gastrointestinal (GI)-tract complications, and cognitive impairments.[8,9] Although some of these clinical features, like myotonia and muscle weakness, are generally shared among DM1 patients, disease manifestation is extremely variable in symptom type, severity, and age of onset.[9] There are multiple contributing factors that cumulatively influence disease manifestation and severity, such as repeat instability, CpG methylation of the locus, presence of repeat interruptions, and, primarily, the length of the repeat.[10–13] With respect to the latter, within one patient, repeat length generally varies among different cell types and tissues and tends to increase with successive generations,[14] thereby enhancing the complexity of DM1 genotypic variability and phenotypic heterogeneity.

Longer inherited $(CTG)_n$ repeat expansions in blood correlate with earlier DM1 onset and more severe symptoms. Additionally, upon transmission, expanded *DMPK* alleles have almost always increased in repeat length, due to somatic instability in the germline. These two facts explain the clinical anticipation observed, which is the decrease in age at onset seen in successive generations.[15–17] However, only few studies have reported on the precise causative relation between

Received 31 January 2025; accepted 30 May 2025;
https://doi.org/10.1016/j.omtm.2025.101502.

[5]These authors contributed equally

[6]These authors contributed equally

Correspondence: Roland Brock, Department of Medical BioSciences, Radboud University Medical Center, PO Box 9101, Nijmegen 6500 HB, the Netherlands.
E-mail: roland.brock@radboudumc.nl

Correspondence: Derick G. Wansink, Department of Medical BioSciences, Radboud University Medical Center, PO Box 9101, Nijmegen 6500 HB, the Netherlands.
E-mail: rick.wansink@radboudumc.nl

© 2025 The Authors. Published by Elsevier Inc. on behalf of The American Society of Gene and Cell Therapy.
This is an open access article under the CC BY license (http://creativecommons.org/licenses/by/4.0/).

repeat length in specific cells and tissues and disease features (reviewed in[18,19]). Moreover, understanding of the relationship between repeat length and the contribution of specific cellular pathomechanisms to the complex DM1 phenotype is essentially lacking. This gap in knowledge renders disease course prediction, as well as the development and design of therapies for individual patients within the highly diverse DM1 patient population, challenging.[20] To date, treatment for DM1 remains merely symptomatic.[21] Recent therapeutic approaches however,[22] particularly those utilizing antisense oligonucleotides (ASOs) that target toxic and expanded *DMPK* transcripts, are proving to be promising in pre-clinical and clinical stage.[23–27] Notably, in the design and patient inclusion of clinical trials, little attention is typically given to the repeat length of individual participants.

Pre-clinical *in vitro* models with relevance to DM1 are needed to both recapitulate repeat-length diversity and improve the evaluation of therapeutic strategies. Although there is a standard DM1 myoblast cell model commonly used in pre-clinical studies,[28] a variety of other DM1 cell cultures and cell types (e.g., fibroblasts, myotubes, and induced pluripotent stem cells [iPSCs], neurons), often primary or immortalized, are available.[29–31] Originating from different tissue types and/or patients, the collective utilization of a subset of these cell cultures brings along variability in differentiation status and genetic background, hindering identification of repeat length-dependent effects in isolation. Recent developments in CRISPR/Cas technology offer possibilities toward the generation of isogenic cell lines bearing different DM1 repeat lengths.[32]

This study tests the hypothesis that length of the $(CTG)_n$ repeat expansion directly correlates with distinct molecular hallmarks of DM1 and thereby influences the effectiveness of antisense oligonucleotides (ASOs) in correcting aberrant splicing in DM1. By using both primary cultures and CRISPR/Cas9-engineered isogenic muscle cell lines with varying repeat lengths, we aim to determine whether repeat length is a critical factor in therapeutic response, independent of genetic background variability.

RESULTS

ASO therapeutic response in a set of primary DM1 cells with different repeat lengths

ASOs are broadly explored as potential drugs in therapeutic interventions for DM1 as they directly target the cause of the disease at the RNA level, namely toxic *DMPK* transcripts bearing extended repeats. Given the repeat-length heterogeneity both among and within DM1 patients, we sought to determine whether therapeutic effects of ASO treatment depended on the repeat length present in treated cells. Two distinct ASOs were used, namely a repeat-targeting blocking-type and a *DMPK*-targeting gapmer ASO, along with their respective controls (Table S1).[33] We obtained primary DM1 myoblast cultures bearing repeat lengths of 800 (pDM800), 1,200 (pDM1200), and >3,000 (pDM>3000) triplets alongside the standard, immortalized human myoblast cell line commonly used as an *in vitro* model for DM1 (iDM2900), bearing a repeat length of 2,900 triplets. Proliferating cell cultures were exposed to 200 nM ASO for 24 h. To avoid introducing an additional variable in our study, we selected this fixed concentration based on previous experiments that indicated that this level generally produces a maximal and robust effect.[33,34] Next, the ASO effect was assessed based on *DMPK* downregulation (using both a 5′ and a 3′ amplicon) and splicing correction of *MBNL1* exon 5 inclusion, *NFIX* exon 7 inclusion, and *CLASP1* exon 19 exclusion. These splicing events are strong predictors of DM1 disease severity.[35] For cellular uptake, ASOs were formulated with the cell-penetrating peptide Pepfect14, which we had previously used for investigation of ASOs in the iDM2900 model and is representative of peptide-based delivery vectors.[34,36,37]

In comparison to the control ASO, the repeat blocker reduced *DMPK* mRNA levels in iDM2900 to 74% and 73% for *DMPK* e1-e2 and e15 (3′), respectively (Figure 1A; Table S2). The primary pDM>3000 and pDM1200 cell cultures showed similar downregulation of *DMPK*, whereas pDM800 only showed a reduction to approximately 90% for both amplicons. Although the binding ability of the repeat blocker is expected to be dependent on the number of binding sites, the effect of the repeat blocker on *DMPK* in these primary cultures did not seem to be influenced by repeat length. The *DMPK* gapmer, which targets expanded and nonexpanded transcripts equally, induced drastic downregulation of *DMPK* in iDM2900 to 19% and 24%. This downregulation was less in pDM>3000 (50% and 56%) and pDM1200 (45% and 41%) and least in pDM800 (69% and 64%) (Figure 1B).

With regard to splicing correction, there was no detectable effect in splicing correction in the primary cultures with either of the ASO types. By comparison, in iDM2900 cells, splicing of *MBNL1* ex 5 and *CLASP1* ex 19 were corrected by both the blocker and gapmer ASO, whereas splicing of *NFIX* ex 7 was significantly corrected by the gapmer only. Interestingly, the DM1-typical missplicing levels of the primary cultures were considerably lower than those of the control ASO-treated iDM2900 cell line (Figure 2), and in some cases, missplicing levels were similar to the ASO-corrected levels achieved in iDM2900 (particularly for *MBNL1* ex 5 and *CLASP1* ex 19). Considering the expansion lengths in these primary myoblast cultures, these observations were unexpected and implied very mild or no DM1 features in these cells that permitted little to no corrective potential for ASO treatment.

Characterization of primary control and DM1 myoblast cultures

Aberrant splicing is generally taken as a proxy for DM1 severity. The low levels thereof in our primary cell cultures and the corresponding lack of ASO responsiveness in splicing correction prompted us to characterize these cell cultures in more detail. A primary myoblast culture from an unaffected individual (pWT5) was taken along as control.

Since *DMPK* transcripts are the causative toxic agent in DM1, we first compared *DMPK* mRNA levels across cell cultures. Relative expression levels were determined as fold changes (using the $2^{-\Delta\Delta Ct}$

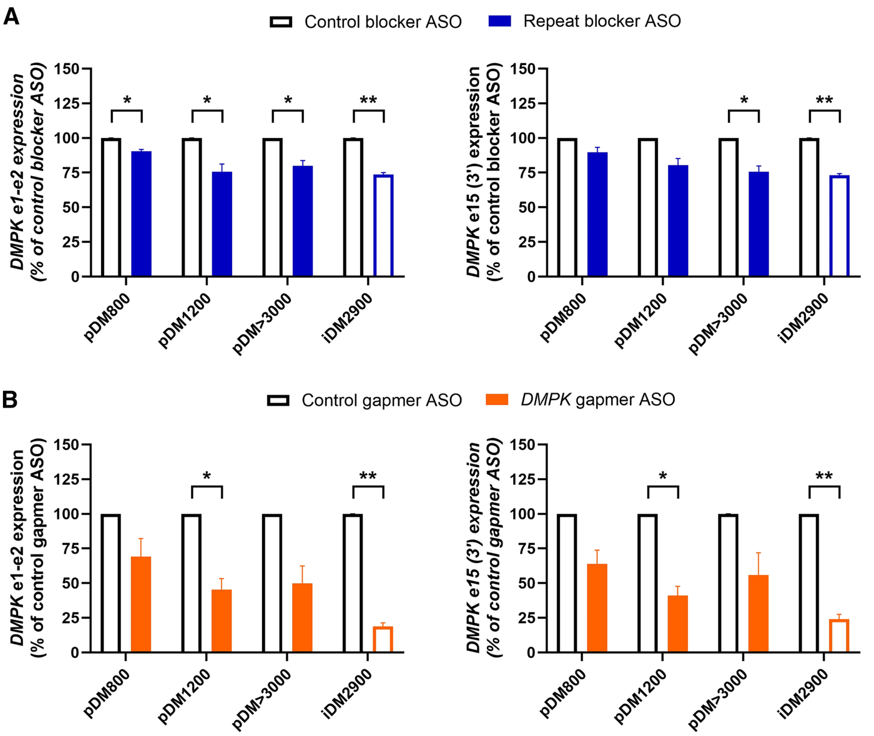

Figure 1. ASO effect on *DMPK* expression in a set of primary DM1 cell cultures

DMPK expression after treatment with 200 nM of (A) the repeat-targeting blocking-type (blue bars) and control (open black bars) ASO or (B) the *DMPK*-targeting gapmer-type (orange bars) and control (open black bars) ASO after 24 h, determined by RT-qPCR for amplicons *DMPK* e1-e2 (left) and e15 (3′) (right). The iDM2900 cell line was included as a reference (blue/orange open bars). Data are presented as mean ± SEM of three independent experiments. A one-sample t test was used to compare the effect of each ASO with its respective control (set at a theoretical 100%) per cell culture. *$p < 0.05$, **$p < 0.01$.

method), with iDM2900 serving as the reference. These values were then converted to percentages, where expression in iDM2900 was defined as 100%. *DMPK* expression in the primary cultures was generally lower than in the iDM2900 line, and cell cultures varied greatly from one another (Figure 3A). Interestingly, pDM>3000, bearing a comparable repeat length to iDM2900, showed only half of the *DMPK* expression as iDM2900. By comparison, pDM1200 showed a relatively high expression (78% and 102% for DMPK e1-e2 and e15 (3′), respectively), relative to iDM2900. In contrast, pDM800 and pWT5 (from an unaffected control) showed an equally low *DMPK* expression of only about 5% of iDM2900 levels. The variability in *DMPK* mRNA levels and its generally low abundance in the primary cells were unrelated to repeat length. These variations are probably related to the tissue origin, genetic background, or passage number and must be considered as variables affecting downstream pathogenic processes, like missplicing.

One may expect that the sequestration of the MBNL1 protein and, as a result, the degree of missplicing in primary cultures is a function of both the length of the $(CTG)_n$ repeat and the expression levels of *DMPK* transcripts. We indeed found that the level of aberrant splicing in our primary cultures was generally lower than in the iDM2900 cell line. This was most evident for *MBNL1* ex 5 and *NFIX* ex 7, where all primary DM1 cells showed a relatively mild level of missplicing (Figure 3B). Moreover, pDM800, the cell culture with the lowest *DMPK* mRNA levels, displayed a similar splicing profile as the pWT5 culture for *MBNL1* ex 5 and *NFIX* ex 7 inclusion, and although the primary cell cultures with longer repeats (pDM1200 and pDM>3000) did show some missplicing, the degree of aberrant splicing was still considerably lower than that of iDM2900. Splicing of *CLASP1* ex 19 showed some correlation for longer repeat lengths (pDM1200, pDM>3000, and iDM2900) but not for pDM800 and/or pWT5.

Uptake of ASOs in this study was mediated by non-covalent complexation with the peptide-based delivery vehicle Pepfect14 into nanoparticles, which are taken up by endocytosis. Cells may differ in their endocytic capacity, thus ASO transfection efficiency could be cell-culture dependent and contribute to the degree of *DMPK* downregulation observed. Therefore, we determined whether the primary cell cultures differed in ASO uptake. For this purpose, we used Cy5-labeled ASOs to image the uptake and nuclear localization of ASOs. Quantification of the mean nuclear fluorescence corresponding to ASO-associated Cy5 signal revealed different distributions of fluorescence intensities in nuclei of primary cells in comparison to iDM2900 (Figure S1A), particularly in the fraction of nuclei with higher fluorescence intensities. The pDM800 and pWT5 cell cultures had a relatively low fraction of nuclei with high fluorescence intensities, whereas pDM1200 had a larger fraction of nuclei with high intensities. Transfection efficiency, indicated by the number of "positive" nuclei (i.e., nuclei exceeding the threshold of background fluorescence), albeit non-significantly due to large variations, differed markedly among the primary cell cultures, with 26%, 14%, 47%, and 33% for pWT5, pDM800, pDM1200, and pDM>3000, respectively (Figure S1B). Here, only the pDM1200 cell culture showed a similar transfection efficiency as the iDM2900 cell line, indicating that for the remaining cell cultures, the poor uptake of ASOs was a potentially contributing factor in the observed low ASO effectiveness.

Generation of an isogenic DM1 myoblast panel with variable repeat lengths

To exclude the above-identified known and other unknown variables in studying the relationship between $(CTG)_n$ repeat length, DM1

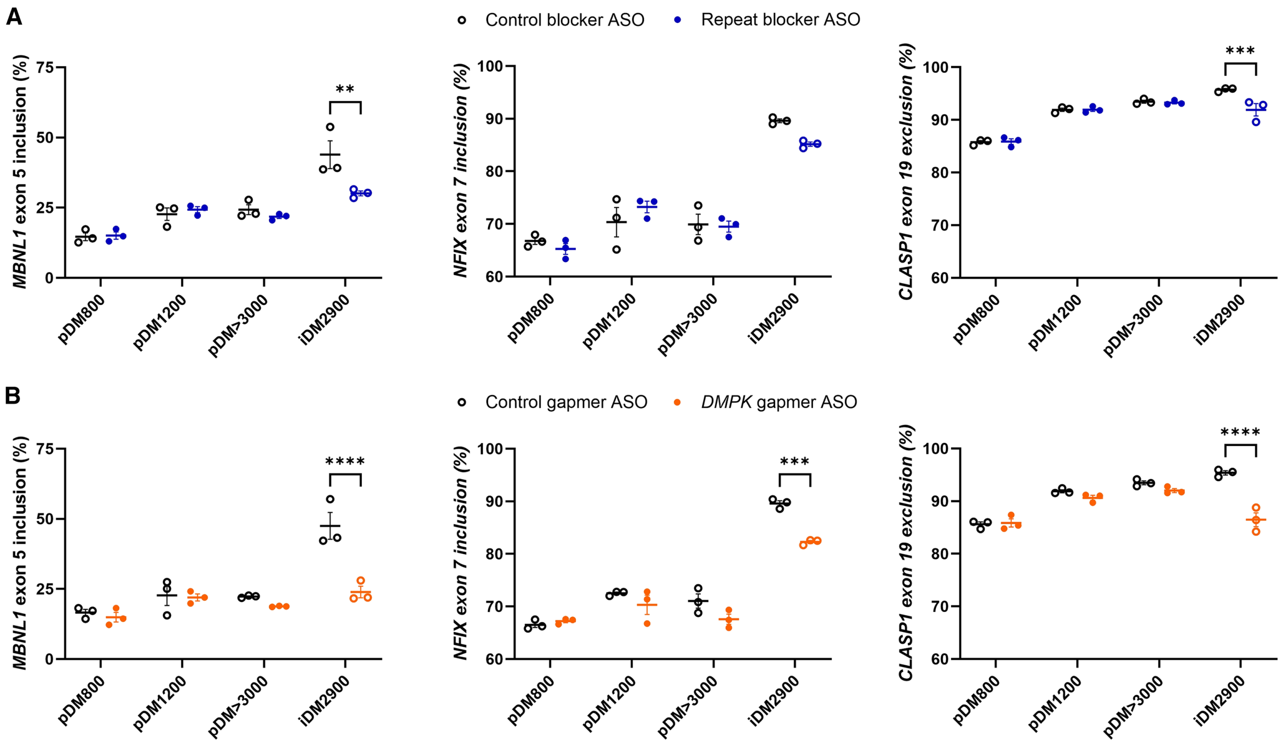

Figure 2. ASO effect on DM1-associated missplicing in primary DM1 cell cultures

Alternative splicing of *MBNL1* ex 5 (left), *NFIX* ex 7 (middle), and *CLASP1* ex 19 (right) after treatment with 200 nM of (A) the repeat-targeting blocking-type (blue circles) and control (open black circles) ASO or (B) the *DMPK*-targeting gapmer-type (orange circles) and control (open black circles) ASO for 24 h, quantified by RT-PCR splicing assays. The iDM2900 cell line was included as a reference. Data are presented as mean ± SEM of three independent experiments. A two-way ANOVA with Šídák post hoc test was used to compare the effect of each ASO with its respective control for each cell culture. $^{**}p < 0.01$. $^{***}p < 0.001$, $^{****}p < 0.0001$.

hallmarks, and ASO efficacy, we applied CRISPR/Cas technology to generate a panel of isogenic cell lines differing only in repeat length. We employed Cas9 nickase (Cas9n), targeted at the repeat, to induce one or more single-stranded cuts, ultimately leading to the loss of a significant number of triplets in the expanded *DMPK* allele (Figure 4A). iDM2900 cells were therefore nucleofected with plasmids expressing Cas9n-T2A-GFP and a $(CAG)_6$ gRNA, followed by selection and cloning of cells from the top 1% highest GFP-expressing fraction using FACS.

To determine the repeat lengths in the isolated clonal lines, we first performed fragment length analysis of digested genomic DNA using Southern blotting with a radioactively labeled repeat probe to preselect candidate lines where the repeat expansion appeared altered (data not shown). Next, Optical Genome Mapping (OGM, Bionano) was performed to determine the repeat length in individual clonal lines.[38] OGM is an amplification-free technology in which high molecular weight DNA is fluorescently labeled at known 6-mer CTTAAG motifs. The labeling pattern is then compared to a given reference. Changes in label pattern and distances between the reference and a given sample, e.g., by the presence of an unstable repeat,[39,40] can easily be detected. The insertion size and thus number of triplets can be calculated by dividing the insertion in base pairs by three (Table S3). We utilized this method to determine the modal repeat length and potential somatic instability in the Cas9n-generated clones and in the iDM2900 at different passage numbers (at the time of nucleofection [p17] and at a later passage [p26]), to be able to account for potential repeat instability during culturing.

Following data acquisition on a Saphyr system, OGM data were analyzed using a so-called Local Guided Assembly (Local-GA), a recently developed algorithm described by van der Sanden et al.[41] (see materials and methods). Reads were also visualized in molecule distance plots to inspect for instability of the repeat expansion (Figure 4B). The original iDM2900 lines at p17 and p26 harbored, as expected, an expanded repeat in the range of 2,775–2,955 triplets (Table S3). Inspection of the molecule distance plots at the different passage numbers showed a highly similar read distribution for both samples, indicating that the repeat expansion in the immortalized myoblasts showed only minor, non-significant changes in length when maintained in culture.

Two of the screened Cas9n-treated clones (not further used in this study) contained a comparable length to iDM2900 (2733 triplets) or a further expanded allele (3418 triplets), which could be explained by a non-hit and/or (in-)correct repair of the nick. Two other clones

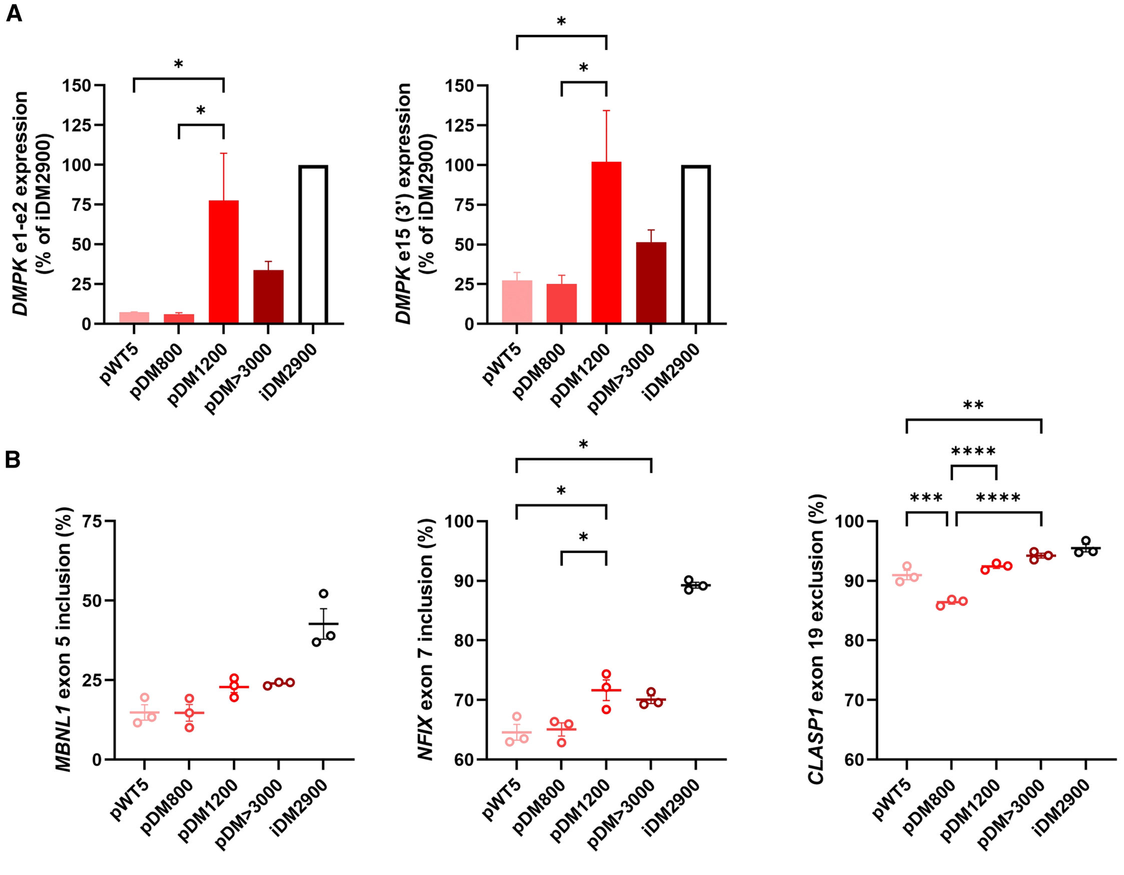

Figure 3. Characterization of primary cultures bearing different repeat lengths

(A) *DMPK* expression of primary cell cultures (light pink to dark red bars) relative to the iDM2900 cell line (open black bar), determined by RT-qPCR for amplicons *DMPK* e1-e2 (left) and e15 (3′) (right). (B) DM1-associated missplicing of *MBNL1* ex 5 (left), *NFIX* ex 7 (middle), and *CLASP1* ex 19 (right), quantified by RT-PCR splicing assays. The iDM2900 cell line was included as a reference. Data are presented as mean ± SEM of three independent experiments. A one-way ANOVA with Tukey post hoc test was used for comparison between all cell cultures. *$p < 0.05$, **$p < 0.01$. ***$p < 0.001$, ****$p < 0.0001$.

exhibited contractions of the repeat with 2,237 (iDM2200) and 1,204 (iDM1200) remaining triplets at the *DMPK* locus. These clones were of particular interest since they probably model milder DM1 features compared to iDM2900. The insertion size of the iDM2200 cell line was manually calculated based on single molecules, since Local-GA was unable to fully model the presence of multiple expanded alleles, as assumed in this cell line based on the molecule distance plot (one for the nonexpanded and two for the expanded allele). Molecules were therefore first ranked based on the insertion size to make a division into molecules covering the nonexpanded allele (upper limit of 800 bp) and expanded alleles. Repeat length was subsequently calculated by averaging the insertion size of all molecules assigned to the expanded allele.

Notably, in all clonal lines, the nonexpanded allele was stable at 13 CTG triplets as quantified by Sanger sequencing. To confirm that the nickase treatment specifically targeted the extreme DM1 repeat expansion only, well-known stable, non-pathogenic CNG-rich repeats in *AR*, *ATN1*, *ATXN1*, *PPP2R2B*, *TBP*, and *TCF4* were screened for off-target activity by PCR amplification and Sanger sequencing and aligned against the background of the iDM2900 cell line (Table S4). All lengths of the six tested microsatellite repeats, each comprising 8–37 triplets, were found to match the iDM2900 genome, including heterozygous alleles. No mutations were detected.

In sum, we generated iDM1200 and iDM2200, two isogenic cell lines derived from iDM2900 with shorter repeat lengths. Together with the in-house generated, iDM2900-derived line lacking the repeat expansion, named here iDM0,[42] we established an isogenic cell panel differing solely in repeat length, allowing us to exclusively assess the effects of repeat length on DM1 features and ASO activity.

Repeat-length dependence of MBNL1 sequestration and DM1-related missplicing

As the primary cells greatly varied in *DMPK* mRNA levels, we first assessed expression levels in the engineered isogenic panel. All lines exhibited equal *DMPK* expression levels (Figure 5A), demonstrating the validity of the panel to address repeat-length dependencies.

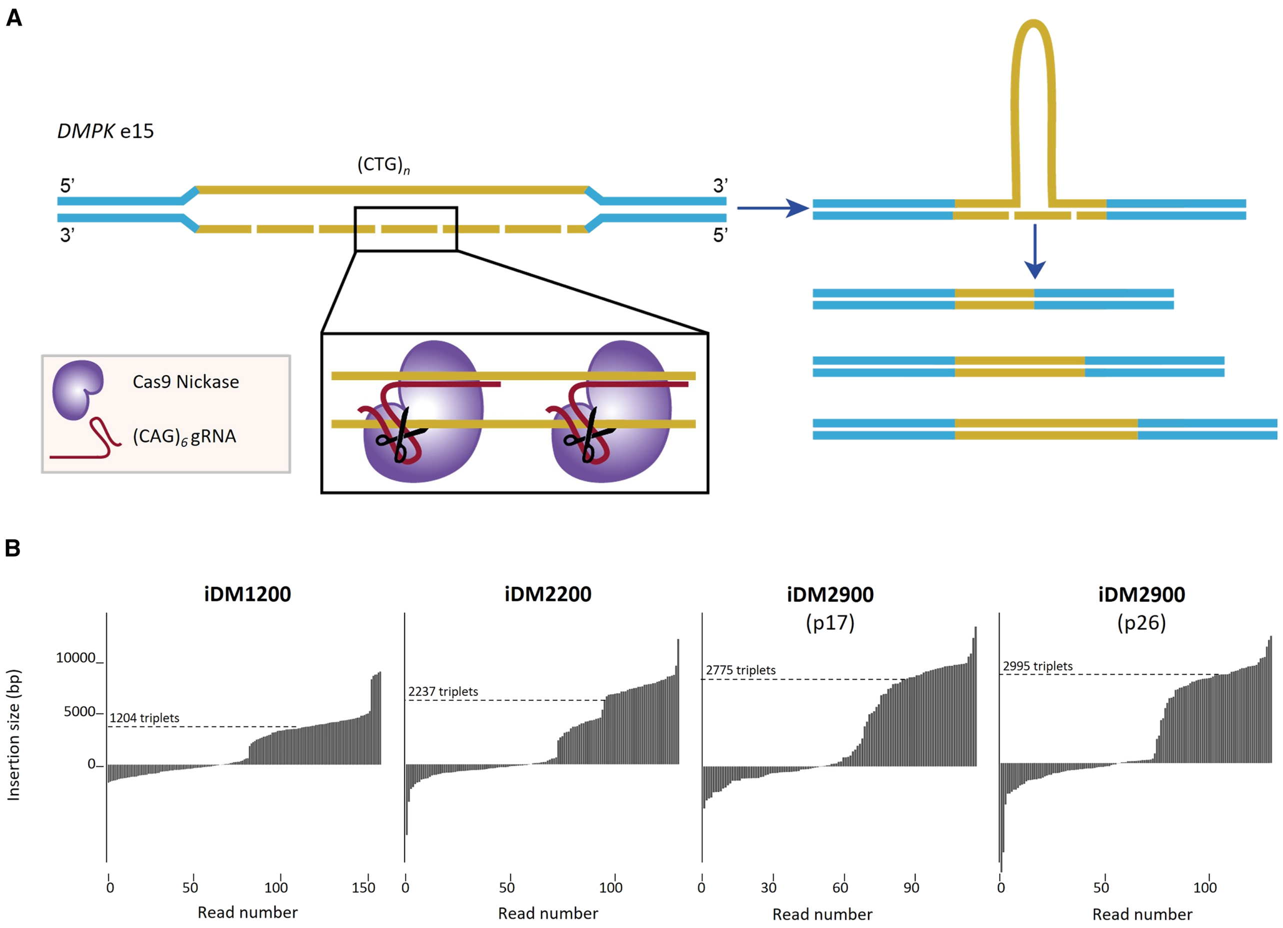

Figure 4. CRISPR/Cas9-nickase-mediated generation of isogenic cell lines bearing different repeat lengths

(A) The DM1-causing repeat expansion in exon 15 of the *DMPK* gene was targeted by Cas9 nickase using a $(CAG)_6$ gRNA. The generation of single-stranded cuts, combined with the cell's DNA repair machinery, ultimately results in an expanded or contracted repeat. iDM2900 cells (p17) were nucleofected with plasmids containing a Cas9n-T2A-GFP and $(CAG)_6$ gRNA and GFP selected by FACS. (B) Determination of repeat length based on optical genome mapping (OGM) of two clones (iDM1200 and iDM2200) and the original cell line at two passage numbers. Molecule distance plots present molecules including the repeat expansion. Insertion size (bp) was determined by the distance between two flanking labels minus the reference length. The number of triplets was calculated by dividing the insertion in base pairs by three. The modal or mean repeat length (dashed line) was rounded to the nearest multiple of 100 and is indicated in the name of the cell line. See the materials and methods section for further explanation of the analysis.

DMPK mRNA levels are therefore no longer a contributing factor to differences in DM1 features between cell lines in the panel.

Splicing in the iDM0 cell line represented the normal (control) level of exon inclusion or exclusion. All three tested alternative splicing events revealed a clear correlation between repeat length and the degree of exon inclusion or exclusion in the isogenic cell panel (Figure 5B). iDM1200, iDM2200, and iDM2900 displayed increasingly more fetal splicing patterns, in line with the consensus of a correlation between repeat length and disease severity. This characteristic was most evident for *MBNL1* ex 5 inclusion and *CLASP1* ex 19 exclusion in the lines with longer expansions (iDM2200 and iDM2900). *NFIX* ex 7 missplicing was less distinct, but also here, a marked trend was observed between repeat length and exon inclusion levels. The isogenic immortalized cell panel thus demonstrates clear evidence of a repeat-length-dependent degree of a fetal transcriptome.

Dysregulation of alternative splicing in DM1 is primarily mediated by reduction of free MBNL1 protein as a result of sequestration by expanded (CUG) motifs.[43,44] To determine whether the observed repeat-length-dependent gradient in missplicing coincides with a change in free nuclear MBNL1 protein in this isogenic cell panel, RNA foci (a hallmark of DM1 cells) were detected by a $(CAG)_6$ probe using RNA fluorescence *in situ* hybridization (FISH), followed by MBNL1 protein staining using immunofluorescence. Fluorescence was measured by automated quantitative image analysis to extract RNA foci, total nuclear MBNL1, and foci-associated MBNL1 signal

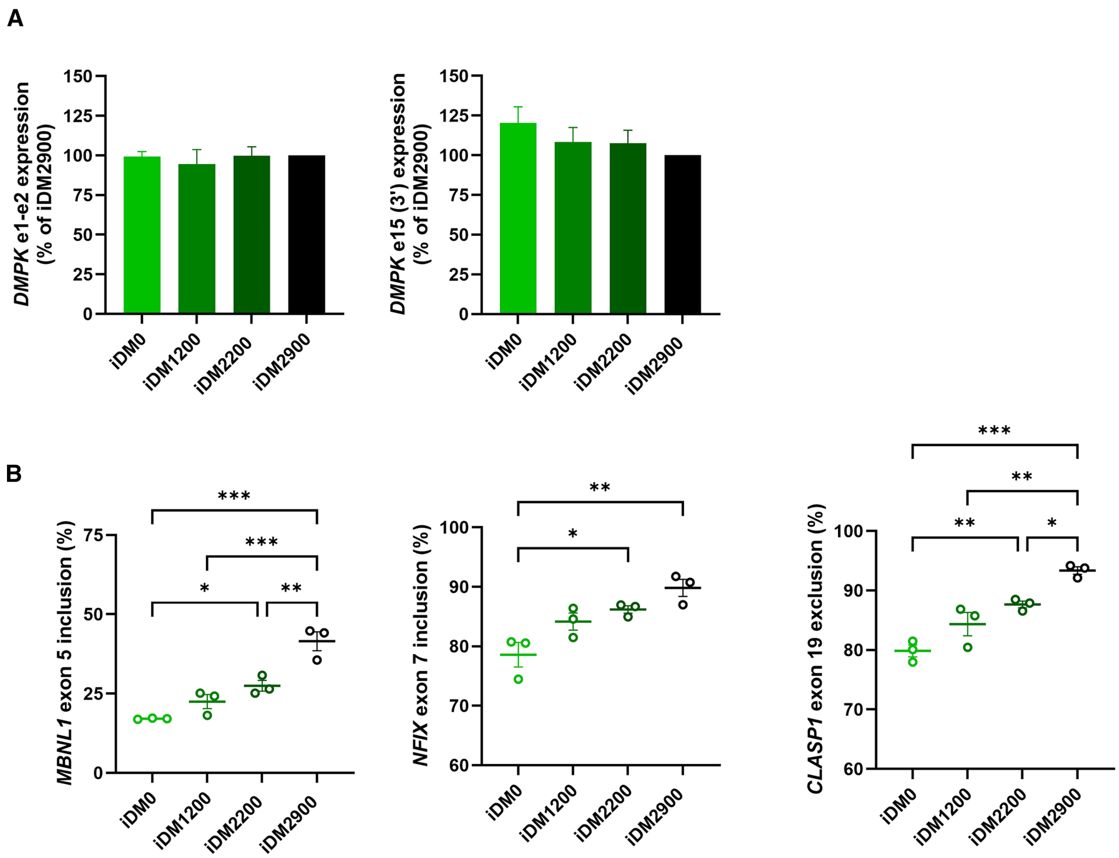

Figure 5. Repeat-length dependence of aberrant splicing in the isogenic DM1 cell panel

(A) *DMPK* expression of the isogenic cell panel, normalized to the iDM2900 cell line, determined by RT-qPCR for amplicons *DMPK* e1e2 and e15 (3′). (B) DM1-associated alternative splicing of *MBNL1* ex 5 (left), *NFIX* ex 7 (middle), and *CLASP1* ex 19 (right), quantified by RT-PCR splicing assays. Data are presented as mean ± SEM of three independent experiments. A one-way ANOVA with Tukey post hoc test was used for comparison between all cell lines. *$p < 0.05$, **$p < 0.01$. ***$p < 0.001$, ****$p < 0.0001$.

from each nucleus (Figure 6A). All isogenic DM1 cell lines contained RNA foci, whereas no foci were detected in iDM0, as reported earlier (Figure 6B). The number of nuclear foci showed a broad distribution, both for the iDM2900 cell line as well as for the newly generated iDM1200 and iDM2200 lines, ranging from only a few to dozens of foci per nucleus. Marginal, but significant, differences were observed between the DM1 cell lines, with iDM2200 showing on average less foci than iDM1200 or iDM2900. More importantly, free nuclear MBNL1 abundance showed a clear trend in relation to repeat length, with longer repeat lengths corresponding to lower levels of available nuclear MBNL1 protein in the cell (Figure 6C). The isogenic cell lines thus demonstrated a clear DM1 pathology with increasing severity as repeat length increased.

Assessment of ASO therapeutic response in the isogenic DM1 cell panel

To evaluate ASO responsiveness for the two types of ASOs exclusively in the context of repeat-length heterogeneity, the effect on *DMPK* downregulation and correction of DM1-associated missplicing were assessed. The repeat blocker led to a downregulation of *DMPK* mRNA to levels ranging from 89% to 69% (between iDM1200, iDM2200, and iDM2900), with increasing repeat length (Figure 7A). As expected, a significant reduction was achieved for iDM2900, and there was no effect for the cell line lacking the repeat expansion (iDM0). For all lines, including iDM0, the *DMPK* gapmer decreased *DMPK* expression to an average level of 26% and 21% for *DMPK* e1-e2 and e15 (3′), respectively (Figure 7B). *DMPK* downregulation was more pronounced than in the primary cultures. Notably, also the transfection efficiencies were higher and more uniform among the isogenic lines (Figure S1B).

As observed earlier, in iDM2900, the repeat blocker led to correction of aberrant splicing of *MBNL1* (Figures 2 and 8A), whereas for iDM2200, two out of three splicing markers were restored toward the normal (nonexpanded control) iDM0 splice pattern. Significant reduction from 26% to 20% in iDM2200 and 37% to 27% in iDM2900 was detected for *MBNL1* ex 5 inclusion, whereas a more nuanced reduction was observed for *NFIX* ex 7 inclusion and *CLASP1* ex 19 exclusion.

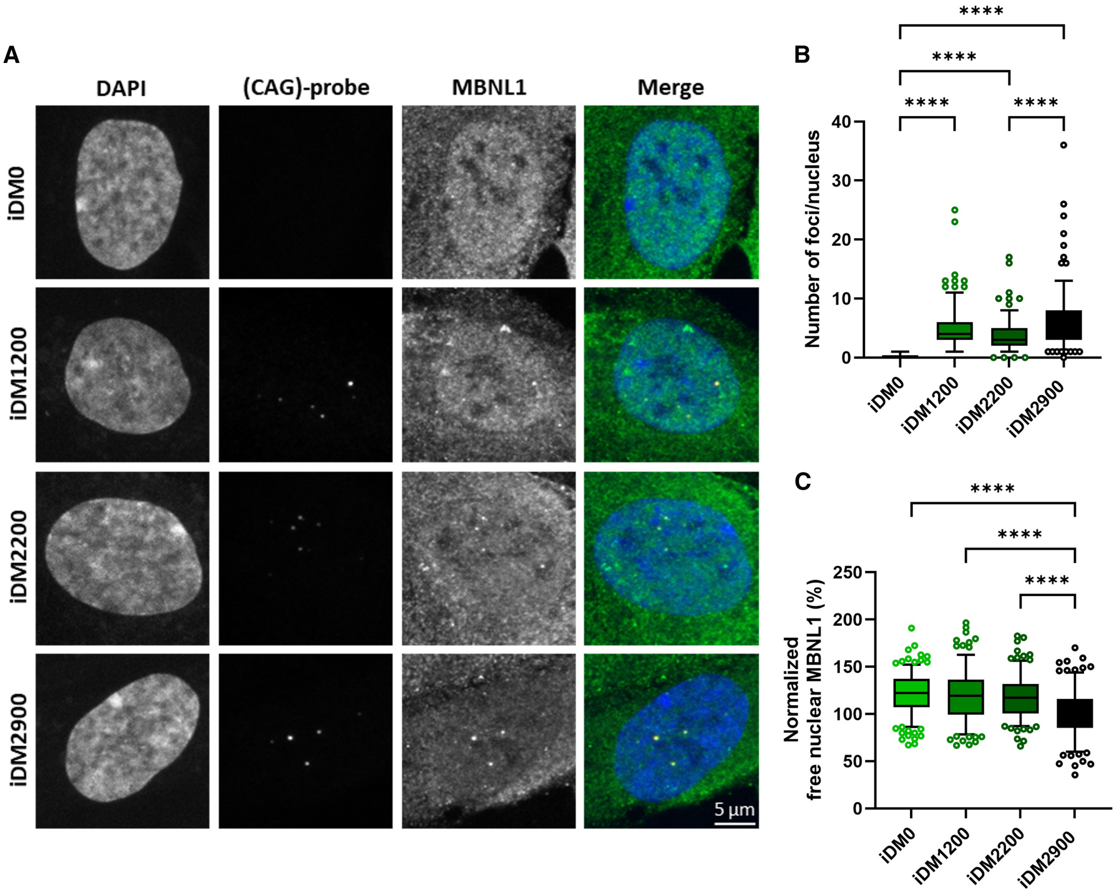

Figure 6. Repeat-length dependence of free nuclear MBNL1 in the isogenic DM1 cell panel

(A) Representative micrographs of nuclei from the isogenic cell panel with visualization of (CUG) RNA foci and MBNL1 protein by FISH and immunofluorescence, respectively. The DAPI and (CAG)-probe channels were corrected for brightness and contrast for an optimal signal-to-noise ratio of micrographs. (B) Average number of foci per nucleus quantified by automated analysis. (C) Free MBNL1 levels within nuclei (normalized to iDM2900), quantified by subtracting the foci-associated MBNL1 signal from the total nuclear MBNL1 signal. Data are presented as box and whisker plots (depicting 5%–95% confidence interval) with single nuclei from three independent experiments, with at least 50 nuclei per experiment. A Kruskal-Wallis (nonparametric) test with Dunn's post hoc test was used for comparison between cell lines. *$p < 0.05$, **$p < 0.01$. ***$p < 0.001$, ****$p < 0.0001$.

The effect on splicing correction by the *DMPK* gapmer was more prominent. For *MBNL1* ex 5 inclusion, the nonexpanded splice pattern was reconstituted in all cases, and for *NFIX* ex 7 inclusion, both iDM1200 and iDM2200 reached splicing levels comparable to those of control cells (Figure 8B). Interestingly, reduction of *CLASP1* ex 19 exclusion even led to an overshoot effect in iDM0, iDM1200, and iDM2200 cell lines.

Although the therapeutic window for splicing correction narrowed in cell lines with shorter repeats (as shown by the splicing levels in control-ASO-treated cell lines in comparison to control levels), it is noteworthy that both ASOs were able to improve the degree of missplicing across all repeat-expanded lines. Specifically, the corrective capacity of the repeat blocker reduced with increasing repeat length, as the splicing levels achieved after ASO treatment increasingly deviated from those of the nonexpanded control cell line. In contrast, the *DMPK* gapmer ASO effectively normalized aberrant splicing levels, leaving minimal residual deviation from the control levels.

DISCUSSION

In this study, we demonstrate that, although susceptible to obstruction by the genetic background, repeat length in DM1 is a major determinant for the degree of aberrant splicing, nuclear MBNL1 levels, and therapeutic effectiveness of ASO strategies.

We initially employed a set of primary myoblast cell cultures for testing of ASO therapy, as these were expected to be better representative for the disease state in patients, in comparison to more standardized and often modified model systems.[28,30,45,46] *DMPK* mRNA levels and transfection efficiencies appeared highly variable among the primary cultures, and more importantly, splicing patterns

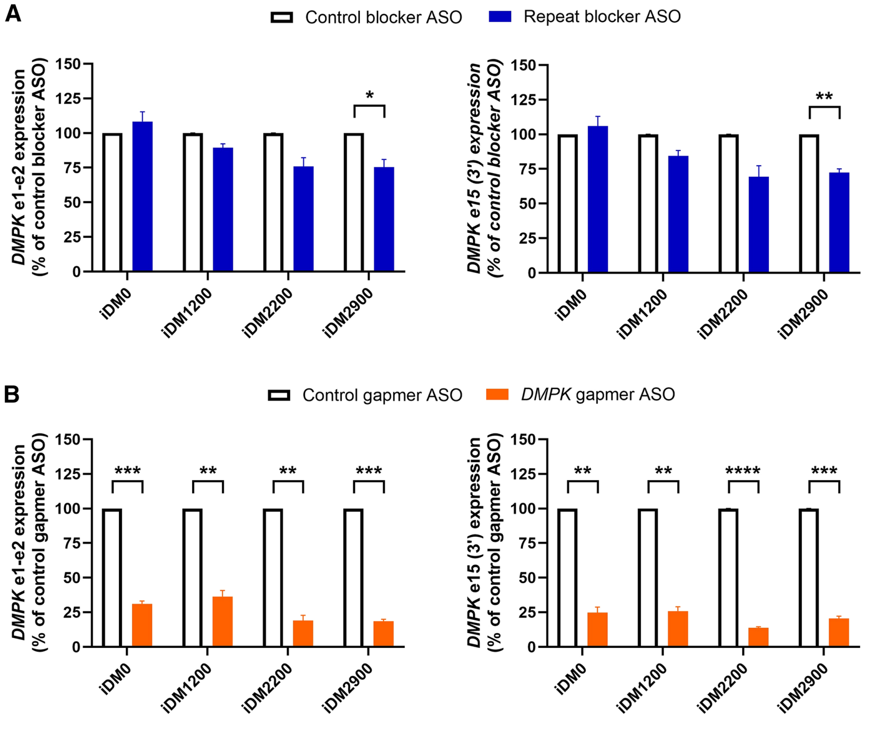

Figure 7. ASO effect on *DMPK* downregulation in isogenic DM1 cell lines

DMPK expression after treatment with 200 nM of (A) the repeat-targeting blocking-type (blue bars) and control (open black bars) ASO or (B) the *DMPK*-targeting gapmer-type (orange bars) and control (open black bars) ASO after 24 h, determined by RT-qPCR for amplicons *DMPK* e1-e2 (left) and e15 (3′) (right). Data are presented as mean ± SEM of three independent experiments. A one-sample t test was used to compare the effect of each ASO with its respective control (set at a theoretical 100%) per cell line. *$p < 0.05$, **$p < 0.01$, ***$p < 0.001$, ****$p < 0.0001$.

were not altered to the degree as would be expected based on repeat length. This finding suggested that multiple factors are confounding disease pathology and possibly also therapeutic responsiveness in DM1. Aspects such as cell origin, muscle type, passage number, and genetic background may, to different degrees, contribute to the disease-related presentation *in vitro*, rendering it difficult to assemble a primary cell panel that recapitulates solely the repeat length heterogeneity in DM1.

The generation of an isogenic cell panel from an immortalized parental cell line unveiled an ideal scenario that allowed us to exclusively assess the influence of repeat length on DM1-related hallmarks *in vitro*, against an otherwise identical genetic background. The addition of the rescued iDM0 cell line as a reference provided us with a clear benchmark, particularly regarding missplicing—the main proxy for DM1 severity at the pre-clinical stage[47,48]—to replicate repeat-length heterogeneity and investigate the correlation between the two. Importantly, this panel had a rather high *DMPK* expression level. With this cell panel, we were able to capture a dependence of repeat length on the severity of pathological processes and ASO response.

The isogenic panel of myoblast lines confirms that repeat length is a key determinant for the degree of aberrant alternative splicing. The significance of particularly *MBNL1* in this has been a main focus point in the DM1 field. Linking the genetic cause of DM1 (i.e., the length of the repeat expansion) to MBNL1 abundance and alternative splicing has proven to be challenging thus far. However, Wagner et al. elegantly demonstrated that MBNL1 protein levels correlate with missplicing of cassette exons (including those in *MBNL1*, *NFIX*, and *CLASP1*).[35] Moreover, the microscopy data in this study provides evidence that the number of triplets influences free MBNL1 protein levels in the nucleus in a dose-dependent manner. Longer repeat expansions potentially sequester more MBNL1 protein, leading to reduced MBNL1 availability. The established isogenic cell panel can thus be utilized as a model system to obtain further insights on expanded CUG RNA-MBNL1 dynamics.

To elucidate the dependence of repeat length more in-depth in the future and to understand for example what the threshold of repeat number may be to trigger specific disease mechanisms, the isogenic cell panel could be expanded by reapplying the highly on-target Cas9-nickase procedure.[32,49] The use of Cas9 nickase facilitates genomic remodeling only at unstable long repeat expansions, in contrast to subjecting the cells to double-strand breaks by regular Cas9, potentially leading to indels or unpredictable off-target effects.

We surmise that the repeat expansion in both the original and the Cas9n-generated clones consists of pure $(CTG/CAG)_n$ triplets. Interruptions, present in 5%–10% of the DM1 population,[20] are thought to slow disease progression by reducing somatic instability in tissues and thus possibly stabilize repeat length *in vitro*.[17,50] Techniques capable of sequencing long repeats of thousands of triplets with high accuracy are under development (e.g., PacBio HiFi sequencing).[51] OGM technology is a sequencing-independent method that is useful for estimating long expansion sizes (>500 bp) without any upper size limit or bias caused by GC-rich regions or repeat tracts.[52] In this context, repeat length analysis of the iDM2200 line (and iDM1200 to a lesser extent) revealed a range in expansion sizes within the cell population, which would likely be undetected by methods like Southern blotting of digested genomic DNA, which has a lower resolution and signal-to-noise ratio. This range (or bimodal pattern) of moderate and long expansion lengths may result from two starting populations upon Cas9 nickase treatment or significant repeat instability during culturing. Given the common and unpredictable repeat-length variability in DM1 patient

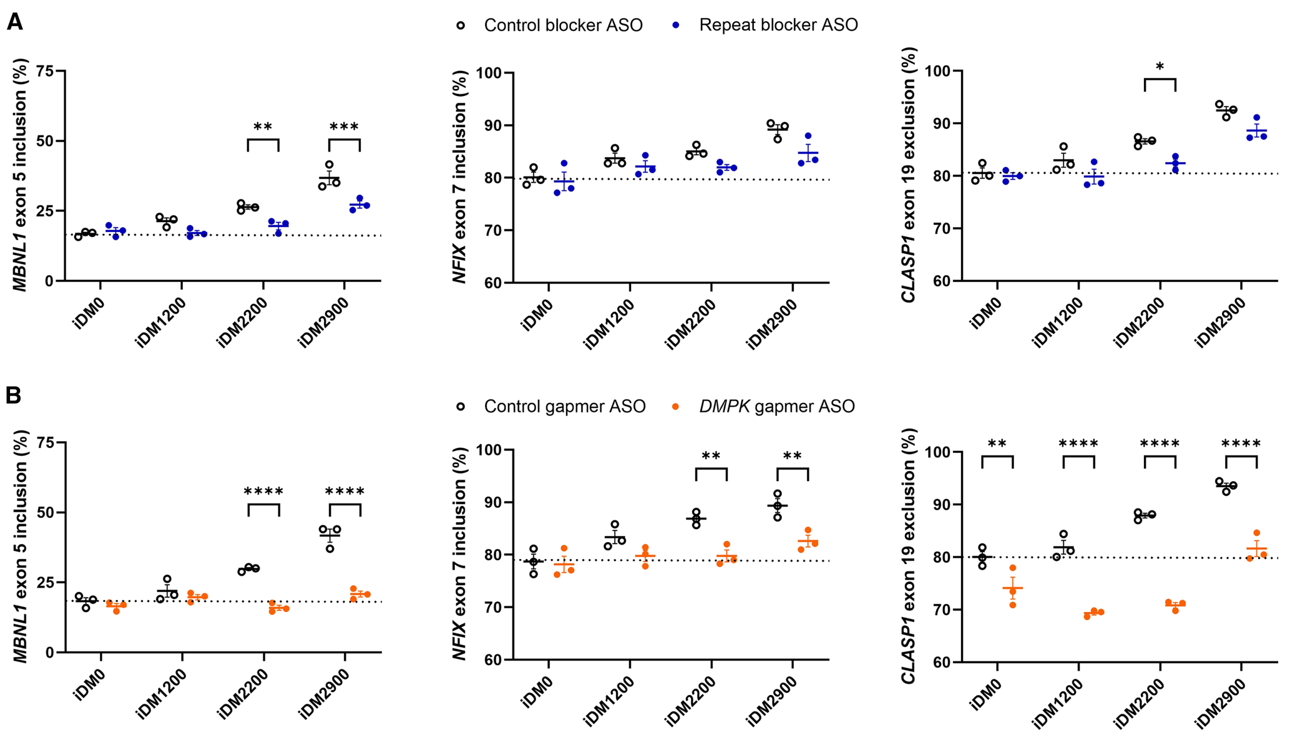

Figure 8. ASO effect on DM1-associated aberrant splicing in isogenic DM1 cell lines

Alternative splicing of *MBNL1* ex 5 (left), *NFIX* ex 7 (middle), and *CLASP1* ex 19 (right) after treatment with 200 nM of (A) the repeat-targeting blocking-type (blue circles) and control (open black circles) ASO or (B) the *DMPK*-targeting gapmer-type (orange circles) and control (open black circles) ASO for 24 h, quantified by RT-PCR splicing assays. Data are presented as mean ± SEM of three independent experiments, and the dotted line indicates the splicing level in the nonexpanded control cell line (iDM0). A two-way ANOVA with Šídák post hoc test was used to compare the effect of each ASO with its respective control for each cell line. *$p < 0.05$, **$p < 0.01$. ***$p < 0.001$, ****$p < 0.0001$.

tissues *in vivo*, we therefore consider the iDM2200 line a true representative of the muscle cell population.

DMPK is highly expressed in skeletal, cardiac, and smooth muscle tissue,[53,54] which likely explains why these tissues are most affected in DM1 patients. In this study, the isogenic cell panel exhibited a relatively high level of *DMPK* mRNA expression compared to the primary cells. The lower levels of *DMPK* in some of the primary cultures may reflect the type of muscle and the embryonic or differentiation state from which they originate or that they obtained *in vitro*.[55,56] These factors, along with repeat length, likely influence and, in this case, mask the extent of aberrant splicing defects in the primary cell cultures. Importantly, repeat length varies between different cells, cell types, and tissues of an individual patient and is generally much longer in muscle and brain than in blood.[57–59] In fact, repeat lengths in affected muscle and brain, potentially in a selected portion of cells, can exceed many thousands of triplets, much more than present in the primary cell collection used here. Therefore, we propose that the DM1 phenotype is likely a function of both the mRNA level of expanded *DMPK* transcripts and the repeat length that these transcripts contain. This notion is consistent with the distinct differences in severity of the phenotypes in transgenic DM1 mouse models like, DMSXL, HSA^{LR}, and *Dmpk* CTG^{480} knockin mice.[60–62]

The therapeutic response to the *DMPK* gapmer was substantial throughout all isogenic cell lines. This ASO showed to be independent of repeat length in downregulating *DMPK* levels and generally also in splicing correction of both *MBNL1* ex 5 inclusion and *NFIX* ex 7 inclusion. The effect on correction of *CLASP1* ex 19 exclusion was less pronounced due to an unexpected overshoot effect in cell lines with shorter repeat lengths (including iDM0). Although we have observed a similar effect using an alternative method to reduce *DMPK* mRNA expression (Ripken et al., unpublished data), the mechanism behind and consequences of this overshoot effect are yet to be determined. We presume that release of MBNL1 protein upon ASO therapy induces a temporary spike in cellular MBNL1 levels that could result in an overshoot effect for more sensitive splicing events.

The repeat blocker, designed to displace MBNL1 proteins from expanded *DMPK* transcripts, showed increasing activity on missplicing correction with longer repeat lengths. The difference in effect size between the two ASOs in the degree of missplicing correction may be attributed to their respective mechanism of action that could result in differences in cellular dynamics at the time of detection. Displacement of a sufficient amount of MBNL1 proteins may require more time before rescue of aberrant splicing can be detected. Moreover, the ability of the repeat blocker to displace MBNL1 proteins can additionally

be influenced by the number of binding sites (i.e., repeat length) and ASO amount available. Nonetheless, in the isogenic cell panel, both ASO types, despite their different mechanisms and behaviors, were able to significantly downregulate *DMPK* and correct missplicing.

While correction of *MBNL1* ex 5 inclusion is a main and robust marker for measuring therapeutic effectiveness, it is noteworthy that missplicing of *MBNL1* ex 5, *NFIX* ex 7, and *CLASP1* ex 19 was not equally rescued after ASO treatment in the isogenic cell panel. This discrepancy is also indicated by the differing degrees of repeat-length dependency upon ASO treatment among the measured splicing events and is likely due to the different ratios and levels of aberrant splicing observed in the isogenic cell lines, as well as the varying sensitivities of the selected splicing markers.[47] Given the increasing number of therapeutics advancing to clinical trials, it is crucial to determine whether treatments will or should be expected to have uniform efficacy across the broad spectrum of DM1 patients and the various tissues affected.

Delivery of ASOs, measured by nuclear ASO distribution and the number of ASO-positive cells, varied among the three primary cultures. This heterogeneity in ASO uptake could be an additional variable influencing the assessment of clinical antisense therapies. The generation of isogenic cell lines also maintained the characteristics of the endocytic pathway of the cells, resulting in a consistent number of transfected cells in the isogenic cultures. Knowing that uptake of nucleic-acid-based therapeutics in muscle tissue is limited,[63] differences in uptake when testing various cell cultures or vehicles should not be overlooked, as these will compromise ASO efficacy.

In summary, primary cells obtained from DM1 biopsies, although they bring us closer to the patient, may not be a fully representative model for studying repeat-length dependence *in vitro*. While genetic and clinical studies have shown correlations between expansion size and disease onset and severity, little has been done to understand the impact of repeat-length heterogeneity on DM1 symptoms and therapeutic interventions. The CRISPR/Cas9-engineered isogenic cell lines provide a platform to investigate the interplay between repeat length and its molecular and cellular consequences in detail, as well as to assess efficacy of therapeutics. Given the current methodologies of pre-clinical *in vitro* screening of ASOs and other therapeutic modalities, candidate selection could be influenced by the cell model used. For the highly variable and heterogeneous DM1 patient population, this generalized approach is probably not ideal. We advocate that both DM1 repeat-length heterogeneity and genetic background should be addressed already at the preclinical stage to better predict therapeutic effects and increase the chances of significant and successful treatment development for the highly diverse DM1 patient population.

MATERIALS AND METHODS

Cell culture

Immortalized human DM1 myoblasts [DM11 cl 5 $(CTG)_{13/2600}$, referred to as iDM2900 in this study] were derived from primary myoblasts from a DM1 patient.[28] This line was previously treated by our group with a dual Cas9 approach to remove the repeat expansion in the diseased allele (CTG_{13}/CTG_{Δ}; iDM0)[42] and was used in this study as an isogenic control. Both the iDM2900 and the iDM0 lines exhibited abnormal hypermethylation of the region 488–777 bp upstream of the repeat (26 CpG sites), which is commonly associated with the congenital form of the disease in patients.[64]

Primary human myoblast cultures pDM800 (nonexpanded allele CTG_{13}), pDM1200 (nonexpanded allele CTG_{5}), and pDM>3000 (nonexpanded allele CTG_{12}) were kind gifts from Drs. Denis Furling and Jack Puymirat.[65–67] Since pDM800 and pDM1200 were collected decades ago, neither their origin nor their passage number could be traced. Repeat lengths were determined at that time via fragment length analysis of digested genomic DNA using Southern blotting with a radioactively labeled probe. pDM>3000 was isolated from a 15-week-old DM1 fetus.[67]

All myoblasts were grown on 0.1% gelatin-coated (G2500; Sigma-Aldrich, Merck KGaA, Darmstadt, Germany) culture plates in proliferation medium consisting of a 1:1 mixture of Skeletal Muscle Cell Growth Medium (PromoCell, Heidelberg, Germany) with 1x GlutaMAX (Gibco, Thermo Fisher Scientific, Landsmeer, the Netherlands) and Ham's F-10 Nutrient Mix with GlutaMAX (Gibco), supplemented with 20% (v/v) HyClone Bovine Growth Serum Supplemented Calf (Cytiva, Medemblik, the Netherlands). Cultures were maintained in a humidified incubator at 37°C and 7.5% CO_2. Medium was refreshed every 2–3 days. For each experiment, cells were seeded at 20,000 cells/cm^2, unless otherwise stated.

Pepfect14-ASO complex formation and transfection

One day prior to transfection, cells were seeded at a density of 20,000 cells/cm^2 in either 12-well plates for RNA isolation and RT-qPCR readouts or 8-well chambered coverslips (Ibidi, Martinsried, Germany) for live cell microscopy. Nanoparticles consisting of ASOs (Table S1) and the cell-penetrating peptide Pepfect14 were formed at a nitrogen to phosphate (N/P) ratio of 3, corresponding to a 9:1 molar ratio of peptide to ASO. Peptide and ASOs were diluted to 20x the final concentration, after which volumes of each were mixed by simultaneous pipetting in a PCR tube.[68] For detection of nuclear uptake of ASOs, a Cy5-labeled $(CAG)_5$ ASO with otherwise identical chemical composition to the repeat-targeting blocking-type $(CAG)_5$ ASO was used. Nanoparticles were then incubated at room temperature for approximately 1 h to allow for stabilization. For transfection, nanoparticles were diluted to a final concentration of 200 nM of the ASO in Skeletal Muscle Cell Growth Medium supplemented with 1x GlutaMAX and 20% (v/v) HyClone Bovine Growth Serum Supplemented Calf. The nanoparticle-containing medium was then added to cells and incubated for 24 h. Cells were then washed 1x with PBS, and phenol-red-free, HEPES-buffered Opti-MEM (Gibco) was added to the IBIDI chambers for live cell imaging or RNA isolation.

RNA isolation and RT-qPCR

Myoblast cultures were washed with 1x PBS before RNA isolation using the Aurum Total RNA mini kit (Bio-Rad, Veenendaal, the

Netherlands) according to manufacturer's protocol, with the addition of pulling cell lysates roughly 10x through a 0.5 mm syringe after lysis to shear genomic DNA. RNA quantity and quality were assessed using a NanoVUE spectrophotometer (GE Healthcare Life Sciences, Nijkerk, the Netherlands) at 260/280 nm and 260/230 nm absorption ratios. cDNA synthesis was performed using the iScript cDNA Synthesis Kit (Bio-Rad) on 500 ng of RNA or the maximum input in a volume of 15 μL in case of low RNA yield. Input was kept constant for each experiment and reverse transcriptase negative controls (RTCs) and a no template control (NTC) were included.

To determine *DMPK* mRNA levels, RT-qPCR was performed in a master mix containing 3 μL of 10x diluted cDNA, 5 μL of iQ SYBR green supermix (Bio-Rad), and 0.4 μM of forward and reverse primer, in a total volume of 10 μL. Samples were run on a CFX96 real-time system (Bio-Rad) using a two-step amplification protocol. *DMPK* expression was determined by amplicons in exon 1–2 and exon 15 (3′) of the repeat expansion (Table S2).[69] *GAPDH* and *HPRT1* were used as reference genes. Melting curves were obtained for all samples to confirm single product amplification.

Alternative splicing of *MBNL1* ex 5, *NFIX* ex 7, and *CLASP1* ex 19 was determined using RT-PCR with primers in adjacent exons of the alternative spliced exons (Table S2). PCR mixes consisted of 1x Q5 reaction buffer (New England Biolabs, Leiden, the Netherlands), 0.2 mM dNTPs, 0.5 μM of forward and reverse primer, 0.4 U Q5 high-fidelity DNA polymerase (New England Biolabs), and 4 μL of 10x diluted cDNA in a total volume of 20 μL. The following program was run on the T100 Thermal Cycler (Bio-Rad): 30 s 98°C, 30x/32x (10 s 98°C, 30 s 71°C, 30 s 72°C), 2 min 72°C, and ∞ 4°C. Detection of PCR products was performed using the QIAxcel Advanced Capillary Electrophoresis System (Qiagen, Germantown, MD, USA) with a DNA high-resolution kit and a 25–500 bp size marker and 15–600 bp alignment marker. RTCs and an NTC were included in each independent experiment.

Live-cell confocal microscopy

After 24 h of incubation with ASO-containing nanoparticles, cells were washed 1x with PBS. To stain nuclei, cells were treated with 5 mg/mL Hoechst 33342 (Thermo Fisher Scientific) in PBS for 5 min, washed with 1x PBS, and maintained in Opti-MEM for imaging. Live-cell confocal microscopy was then performed using a Leica TCS SP8 at 37°C (Leica Microsystems, Buffalo Grove, IL, USA), equipped with a 63x, 1.2 NA water immersion objective. Fluorescence was excited at 405 nm (Hoechst 33342) and 647 nm (Cy5), and emission was collected between 420 and 515 nm (Hoechst 33342) and 655–710 nm (Cy5).

To determine the percentage of cells containing a Cy5-positive nucleus, nuclear masks were made based on the Hoechst 33342 staining. The mean nuclear Cy5-fluorescence and standard deviation (SD) were measured for each nucleus in the field of view. Nuclei were considered positive when the mean Cy5-fluorescence intensity was larger than the SD in the respective area.[70] Areas that showed highly fluorescent signals from other focal planes than the nucleus were distinguished from nuclei with a homogeneous Cy5-fluorescence distribution. At least 50 nuclei per sample were measured for each of the three independent uptake experiments.

Constructs and design of CRISPR systems

The Cas9n approach was based on a previously published protocol.[32] The pSpCas9n(BB)-2A-GFP (PX461) construct was expressed by plasmid #48140 (Addgene, Watertown, MA, USA).[71] The $(CAG)_6$ gRNA was expressed by plasmid #41824 (Addgene) in which two complementary oligos forming the 18 nt gRNA sequence were inserted using the Gibson Assembly kit (New England Biolabs). Correct insertion was verified using in-house Sanger sequencing.

Generation of isogenic cell lines

iDM2900 immortalized myoblasts (p17) were nucleofected using the Neon transfection system (Invitrogen, Thermo Fisher Scientific). In brief, sub-confluent myoblasts were trypsinized, and $1x10^6$ cells were used per transfection. Cells were subsequently washed with 1x PBS and pelleted to enable resuspension of $1x10^6$ cells in 115 μL of R transfection buffer, containing 5 μg of 1:1 (w/w) Cas9 nickase and $(CAG)_6$ gRNA plasmids. Per transfection, 100 μL of cell suspension was loaded into a Neon reaction tip and subjected to one pulse of 1,350 V for 30 ms. Cells were immediately transferred to pre-warmed 0.1% gelatin-coated 6-well plates containing proliferation medium. Cells were left to adhere for 24 h in the incubator. Then, the top 1% GFP-expressing cells were selected by FACS and sorted either single cell or 10 cells/well to increase survival rate. Cells were kept in 1:1 fresh and conditioned medium with 0.5% (v/v) antibiotic-antimycotic (Gibco) until cultures were sufficiently confluent for passaging from a 96-well plate to a 48-well plate. After this step, standard proliferation medium was used. Passage number was counted as px+1 onward.

Optical genome mapping and off-target analysis

Myoblasts were expanded up to $1.5x10^6$ cells per cell line, trypsinized, washed with 1x PBS, and frozen at −70°C as dry cell pellets. Two passages (p17 and p26) were taken along for the DM1 original cell line (iDM2900) to determine repeat (in)stability during culturing. The newly generated isogenic cell lines were screened by OGM between px+4 and px+7, using a published protocol.[72] The length of the variant call was determined using the Local Guided Assembly (Local-GA) workflow, in which consensus maps were generated based on individual molecules covering the *DMPK* gene[38,41] or manually calculated. In the latter, all molecules containing the *DMPK* region were ranked on insertion size. Nonexpanded and expanded alleles were separated based on a sizing cutoff of 800 bp. Size of the expanded allele was subsequently calculated as the average insertion size of molecules assigned to the expanded allele.

Off-target analysis was performed on DNA isolated by a standard proteinase K and ethanol precipitation protocol. Amplicons containing the selected microsatellites were sequenced in-house and verified for the proper repeat sequence (Table S3).

RNA fluorescence *in situ* hybridization and immunofluorescence

Myoblasts were seeded on 0.1% gelatin-coated 10 mm coverslips at 15,000 cells/cm^2. After 24 h, cells were washed with 1x PBS and fixed with 2% PFA in 0.1 M phosphate buffer. Samples were washed 3x with 1x PBS and permeabilized with ice-cold 70% (v/v) ethanol for 1 h at 4°C. Subsequently, the Stellaris RNA FISH kit (LGC Biosearch Technologies, Petaluma, CA) was used with an AF647-labeled $(CAG)_6C$ LNA probe (Exiqon, Vedbaek, Denmark) according to manufacturer's protocol to stain $(CTG)_n$ foci. After the final wash with buffer B, immunofluorescence was performed for MBNL1 protein using a 1:100 dilution of an in-house purified mouse anti-MBNL1 antibody (Developmental Studies Hybridoma Bank). Samples were washed 3x with 1x PBS, permeabilized in 0.1% Triton X-100 (v/v; Sigma-Aldrich) in PBS, and incubated for 1 h at room temperate in blocking buffer consisting of 3% BSA (w/v; Sigma-Aldrich) and 0.1% glycine (w/v; Sigma-Aldrich) in PBS. Primary antibody in blocking buffer was added overnight at 4°C. Samples were washed 3x with 1x PBS, after which cells were incubated for 1 h with 2 μg/mL of an Alexa-Fluor-488-labeled secondary goat anti-mouse antibody (Invitrogen) and 1 μg/mL DAPI (Sigma-Aldrich) for 1 h at room temperate, protected from light. After washing with 1x PBS, coverslips were dehydrated using ethanol and mounted onto object glasses using Mowiol. Confocal microscopy was performed using a Zeiss LSM900 (Carl Zeiss, Jena, Germany) with a 63x, 1.4 NA oil immersion objective. z stack images at 1 μm intervals were taken in which fluorescence was excited at 405 nm (DAPI), 488 nm (Alexa Fluor 488), and 633 nm (AF647), and emission was collected between 400 and 560 nm (DAPI), 490 and 620 nm (AF488), and 640 and 700 nm (AF647).

For quantification of the number of foci and MBNL1 protein abundance, slices from z stack images were combined in maximum intensity projections. Nuclear masks were then created based on DAPI staining, and nuclear foci were counted using the "Find Maxima" function. Nuclear MBNL1 protein was determined by first measuring the total MBNL1 gray values within the nuclear masks followed by measurement of the MBNL1 gray values within 9x9 pixels surrounding each focus and representing the amount of MBNL1 protein sequestered in foci. Free nuclear MBNL1 protein was subsequently calculated by subtracting the MBNL1 gray values in all foci of a single nucleus from the total MBNL1 signal in the same nucleus. For visualization, gray values were transformed to mean gray value (corrected for area) and normalized per independent experiment to iDM2900. In all cases, at least 50 nuclei were measured per condition and per independent replicate.

Data analysis and statistics

Statistics was performed using the GraphPad Prism software (GraphPad Software, La Jolla, CA). Data depicted in graphs are from three independent experiments (each with two technical replicates) and represent the mean ± standard error of the mean (SEM), unless otherwise stated. Corresponding statistical tests are specified in the figure legends. If significant, p values are reported as $^*p < 0.05$, $^{**}p < 0.01$, $^{***}p < 0.001$, or $^{****}p < 0.0001$.

RT-qPCR data was analyzed using the Bio-Rad CFX Manager software (Bio-Rad). Relative mRNA levels were calculated using the ΔΔCt method.[73] The relative abundance of the different splicing isoforms from RT-PCR were determined by the peak calling function of the QIAxcel ScreenGel software (Qiagen, Germantown, MD, USA). The molarity of DM1-dominant isoforms (*MBNL1* ex 5 and *NFIX* ex 7 inclusion and *CLASP1* ex 19 exclusion) was divided by the sum of the molarities of all detected isoforms and expressed as a percentage.

All microscopy images were processed using the open-source FIJI software (accessible at https://imagej.net/Fiji).[74]

DATA AVAILABILITY

All unprocessed and normalized data from this study are available upon request from the corresponding authors.

ACKNOWLEDGMENTS

We kindly thank Dr. Denis Furling and Dr. Vincent Mouly (Institut de Myologie, Paris, France) for providing the immortalized DM1 myoblast cell line (iDM2900), Dr. Denis Furling and Dr. Jack Puymirat for the primary cultures that were used in this study, Eveline Kamping for providing technical OGM support, and Walther van den Broek for technical support. We thank the Radboudumc Technology Center Microscopy for use of their microscopy facilities. This work was funded by the Prinses Beatrix Spierfonds (grant numbers W.OR18–06 and W.OR19-07).

AUTHOR CONTRIBUTIONS

Conceptualization, R.B. and D.G.W.; investigation, N.E.B., L.R., and M.W.; validation, N.E.B. and L.R.; formal analysis, N.E.B., L.R., B.v.d.S., K.N. and A.H.; writing—original draft, N.E.B. and L.R.; writing—review and editing, N.E.B., L.R., R.B., and D.G.W.; visualization, N.E.B. and L.R.; supervision, R.B. and D.G.W.

DECLARATION OF INTERESTS

D.G.W. is inventor on patents involving ASOs for the treatment of myotonic dystrophy type 1.

SUPPLEMENTAL INFORMATION

Supplemental information can be found online at https://doi.org/10.1016/j.omtm.2025.101502.

REFERENCES

1. Johnson, N.E., Butterfield, R.J., Mayne, K., Newcomb, T., Imburgia, C., Dunn, D., Duval, B., Feldkamp, M.L., and Weiss, R.B. (2021). Population-Based Prevalence of Myotonic Dystrophy Type 1 Using Genetic Analysis of Statewide Blood Screening Program. Neurology *96*, e1045–e1053. https://doi.org/10.1212/wnl.0000000000011425.
2. Brook, J.D., McCurrach, M.E., Harley, H.G., Buckler, A.J., Church, D., Aburatani, H., Hunter, K., Stanton, V.P., Thirion, J.P., Hudson, T., et al. (1992). Molecular basis of myotonic dystrophy: expansion of a trinucleotide (CTG) repeat at the 3' end of a transcript encoding a protein kinase family member. Cell *68*, 799–808. https://doi.org/10.1016/0092-8674(92)90154-5.
3. Charlet-B, N., Savkur, R.S., Singh, G., Philips, A.V., Grice, E.A., and Cooper, T.A. (2002). Loss of the muscle-specific chloride channel in type 1 myotonic dystrophy due to misregulated alternative splicing. Mol. Cell *10*, 45–53. https://doi.org/10.1016/s1097-2765(02)00572-5.
4. Fugier, C., Klein, A.F., Hammer, C., Vassilopoulos, S., Ivarsson, Y., Toussaint, A., Tosch, V., Vignaud, A., Ferry, A., Messaddeq, N., et al. (2011). Misregulated alternative splicing of BIN1 is associated with T tubule alterations and muscle weakness in myotonic dystrophy. Nat. Med. *17*, 720–725. https://doi.org/10.1038/nm.2374.
5. Du, H., Cline, M.S., Osborne, R.J., Tuttle, D.L., Clark, T.A., Donohue, J.P., Hall, M. P., Shiue, L., Swanson, M.S., Thornton, C.A., and Ares, M., Jr. (2010). Aberrant alternative splicing and extracellular matrix gene expression in mouse models of

myotonic dystrophy. Nat. Struct. Mol. Biol. *17*, 187–193. https://doi.org/10.1038/nsmb.1720.

6. Gates, D.P., Coonrod, L.A., and Berglund, J.A. (2011). Autoregulated splicing of muscleblind-like 1 (MBNL1) Pre-mRNA. J. Biol. Chem. *286*, 34224–34233. https://doi.org/10.1074/jbc.M111.236547.

7. López-Martínez, A., Soblechero-Martín, P., de-la-Puente-Ovejero, L., Nogales-Gadea, G., and Arechavala-Gomeza, V. (2020). An Overview of Alternative Splicing Defects Implicated in Myotonic Dystrophy Type I. Genes *11*. https://doi.org/10.3390/genes11091109.

8. Wenninger, S., Montagnese, F., and Schoser, B. (2018). Core Clinical Phenotypes in Myotonic Dystrophies. Front. Neurol. *9*, 303. https://doi.org/10.3389/fneur.2018.00303.

9. Meola, G., and Cardani, R. (2015). Myotonic dystrophies: An update on clinical aspects, genetic, pathology, and molecular pathomechanisms. Biochim. Biophys. Acta *1852*, 594–606. https://doi.org/10.1016/j.bbadis.2014.05.019.

10. Morales, F., Corrales, E., Zhang, B., Vásquez, M., Santamaría-Ulloa, C., Quesada, H., Sirito, M., Estecio, M.R., Monckton, D.G., and Krahe, R. (2021). Myotonic dystrophy type 1 (DM1) clinical subtypes and CTCF site methylation status flanking the CTG expansion are mutant allele length-dependent. Hum. Mol. Genet. *31*, 262–274. https://doi.org/10.1093/hmg/ddab243.

11. Cumming, S.A., Jimenez-Moreno, C., Okkersen, K., Wenninger, S., Daidj, F., Hogarth, F., Littleford, R., Gorman, G., Bassez, G., Schoser, B., et al. (2019). Genetic determinants of disease severity in the myotonic dystrophy type 1 OPTIMISTIC cohort. Neurology *93*, e995–e1009. https://doi.org/10.1212/wnl.0000000000008056.

12. Morales, F., Couto, J.M., Higham, C.F., Hogg, G., Cuenca, P., Braida, C., Wilson, R. H., Adam, B., del Valle, G., Brian, R., et al. (2012). Somatic instability of the expanded CTG triplet repeat in myotonic dystrophy type 1 is a heritable quantitative trait and modifier of disease severity. Hum. Mol. Genet. *21*, 3558–3567. https://doi.org/10.1093/hmg/dds185.

13. Miller, J.N., van der Plas, E., Hamilton, M., Koscik, T.R., Gutmann, L., Cumming, S. A., Monckton, D.G., and Nopoulos, P.C. (2020). Variant repeats within the DMPK CTG expansion protect function in myotonic dystrophy type 1. Neurol. Genet. *6*, e504. https://doi.org/10.1212/nxg.0000000000000504.

14. Morales, F., Vásquez, M., Corrales, E., Vindas-Smith, R., Santamaría-Ulloa, C., Zhang, B., Sirito, M., Estecio, M.R., Krahe, R., and Monckton, D.G. (2020). Longitudinal increases in somatic mosaicism of the expanded CTG repeat in myotonic dystrophy type 1 are associated with variation in age-at-onset. Hum. Mol. Genet. *29*, 2496–2507. https://doi.org/10.1093/hmg/ddaa123.

15. Logigian, E.L., Moxley, R.T., 4th, Blood, C.L., Barbieri, C.A., Martens, W.B., Wiegner, A.W., Thornton, C.A., and Moxley, R.T., 3rd (2004). Leukocyte CTG repeat length correlates with severity of myotonia in myotonic dystrophy type 1. Neurology *62*, 1081–1089. https://doi.org/10.1212/01.wnl.0000118206.49652.a3.

16. Arsenault, M.E., Prévost, C., Lescault, A., Laberge, C., Puymirat, J., and Mathieu, J. (2006). Clinical characteristics of myotonic dystrophy type 1 patients with small CTG expansions. Neurology *66*, 1248–1250. https://doi.org/10.1212/01.wnl.0000208513.48550.08.

17. Overend, G., Légaré, C., Mathieu, J., Bouchard, L., Gagnon, C., and Monckton, D.G. (2019). Allele length of the DMPK CTG repeat is a predictor of progressive myotonic dystrophy type 1 phenotypes. Hum. Mol. Genet. *28*, 2245–2254. https://doi.org/10.1093/hmg/ddz055.

18. Yum, K., Wang, E.T., and Kalsotra, A. (2017). Myotonic dystrophy: disease repeat range, penetrance, age of onset, and relationship between repeat size and phenotypes. Curr. Opin. Genet. Dev. *44*, 30–37. https://doi.org/10.1016/j.gde.2017.01.007.

19. de Pontual, L., and Tomé, S. (2022). Overview of the Complex Relationship between Epigenetics Markers, CTG Repeat Instability and Symptoms in Myotonic Dystrophy Type 1. Int. J. Mol. Sci. *23*, 3477. https://doi.org/10.3390/ijms23073477.

20. Tomé, S., and Gourdon, G. (2020). DM1 Phenotype Variability and Triplet Repeat Instability: Challenges in the Development of New Therapies. Int. J. Mol. Sci. *21*, 457. https://doi.org/10.3390/ijms21020457.

21. Ivanovic, V., Meola, G., Vukojevic, Z., and Peric, S. (2023). Update on therapy for Myotonic dystrophy type 1. Curr. Treat. Options Neurol. 25, 261–279.

22. Izzo, M., Battistini, J., Provenzano, C., Martelli, F., Cardinali, B., and Falcone, G. (2022). Molecular Therapies for Myotonic Dystrophy Type 1: From Small Drugs to Gene Editing. Int. J. Mol. Sci. *23*, 4622. https://doi.org/10.3390/ijms23094622.

23. Ait Benichou, S., Jauvin, D., De Serres-Bérard, T., Pierre, M., Ling, K.K., Bennett, C. F., Rigo, F., Gourdon, G., Chahine, M., and Puymirat, J. (2022). Antisense oligonucleotides as a potential treatment for brain deficits observed in myotonic dystrophy type 1. Gene Ther. *29*, 698–709. https://doi.org/10.1038/s41434-022-00316-7.

24. Thornton, C.A., Moxley, R.T., 3rd, Eichinger, K., Heatwole, C., Mignon, L., Arnold, W.D., Ashizawa, T., Day, J.W., Dent, G., Tanner, M.K., et al. (2023). Antisense oligonucleotide targeting DMPK in patients with myotonic dystrophy type 1: a multicentre, randomised, dose-escalation, placebo-controlled, phase 1/2a trial. Lancet Neurol. *22*, 218–228. https://doi.org/10.1016/s1474-4422(23)00001-7.

25. Fiszer, A. (2023). All roads lead to cure: Diversity of oligonucleotides in DM1 therapy. Mol. Ther. Nucleic Acids *32*, 898–899. https://doi.org/10.1016/j.omtn.2023.05.012.

26. Pascual-Gilabert, M., Artero, R., and López-Castel, A. (2023). The myotonic dystrophy type 1 drug development pipeline: 2022 edition. Drug Discov. Today *28*, 103489. https://doi.org/10.1016/j.drudis.2023.103489.

27. Klein, A.F., Varela, M.A., Arandel, L., Holland, A., Naouar, N., Arzumanov, A., Seoane, D., Revillod, L., Bassez, G., Ferry, A., et al. (2019). Peptide-conjugated oligonucleotides evoke long-lasting myotonic dystrophy correction in patient-derived cells and mice. J. Clin. Investig. *129*, 4739–4744. https://doi.org/10.1172/jci128205.

28. Arandel, L., Polay Espinoza, M., Matloka, M., Bazinet, A., De Dea Diniz, D., Naouar, N., Rau, F., Jollet, A., Edom-Vovard, F., Mamchaoui, K., et al. (2017). Immortalized human myotonic dystrophy muscle cell lines to assess therapeutic compounds. Dis. Model. Mech. *10*, 487–497. https://doi.org/10.1242/dmm.027367.

29. Matloka, M., Klein, A.F., Rau, F., and Furling, D. (2018). Cells of Matter-In Vitro Models for Myotonic Dystrophy. Front. Neurol. *9*, 361. https://doi.org/10.3389/fneur.2018.00361.

30. Pantic, B., Borgia, D., Giunco, S., Malena, A., Kiyono, T., Salvatori, S., De Rossi, A., Giardina, E., Sangiuolo, F., Pegoraro, E., et al. (2016). Reliable and versatile immortal muscle cell models from healthy and myotonic dystrophy type 1 primary human myoblasts. Exp. Cell Res. *342*, 39–51. https://doi.org/10.1016/j.yexcr.2016.02.013.

31. Núñez-Manchón, J., Capó, J., Martínez-Piñeiro, A., Juanola, E., Pesovic, J., Mosqueira-Martín, L., González-Imaz, K., Maestre-Mora, P., Odria, R., Cerro-Herreros, E., et al. (2024). Immortalized human myotonic dystrophy type 1 muscle cell lines to address patient heterogeneity. iScience *27*, 109930. https://doi.org/10.1016/j.isci.2024.109930.

32. Cinesi, C., Aeschbach, L., Yang, B., and Dion, V. (2016). Contracting CAG/CTG repeats using the CRISPR-Cas9 nickase. Nat. Commun. *7*, 13272. https://doi.org/10.1038/ncomms13272.

33. El Boujnouni, N., van der Bent, M.L., Willemse, M., t Hoen, P.A.C., Brock, R., and Wansink, D.G. (2023). Block or degrade? Balancing on- and off-target effects of antisense strategies against transcripts with expanded triplet repeats in DM1. Mol. Ther. Nucleic Acids *32*, 622–636. https://doi.org/10.1016/j.omtn.2023.04.010.

34. van der Bent, M.L., Paulino da Silva Filho, O., Willemse, M., Hällbrink, M., Wansink, D.G., and Brock, R. (2019). The nuclear concentration required for antisense oligonucleotide activity in myotonic dystrophy cells. FASEB J. *33*, 11314–11325. https://doi.org/10.1096/fj.201900263R.

35. Wagner, S.D., Struck, A.J., Gupta, R., Farnsworth, D.R., Mahady, A.E., Eichinger, K., Thornton, C.A., Wang, E.T., and Berglund, J.A. (2016). Dose-Dependent Regulation of Alternative Splicing by MBNL Proteins Reveals Biomarkers for Myotonic Dystrophy. PLoS Genet. *12*, e1006316. https://doi.org/10.1371/journal.pgen.1006316.

36. Ezzat, K., Andaloussi, S.E.L., Zaghloul, E.M., Lehto, T., Lindberg, S., Moreno, P.M. D., Viola, J.R., Magdy, T., Abdo, R., Guterstam, P., et al. (2011). PepFect 14, a novel cell-penetrating peptide for oligonucleotide delivery in solution and as solid formulation. Nucleic Acids Res. *39*, 5284–5298. https://doi.org/10.1093/nar/gkr072.

37. Periyasamy, K., Maloverjan, M., Biswas, A., Remm, A., Pook, M., Rebane, A., and Pooga, M. (2023). PepFect14 mediates the delivery of mRNA into human primary keratinocytes and in vivo. Front. Pharmacol. *14*, 1219761. https://doi.org/10.3389/fphar.2023.1219761.

38. van der Sanden, B., Neveling, K., Shukor, S., Gallagher, M.D., Lee, J., Burke, S.L., Pennings, M., van Beek, R., Oorsprong, M., Kater-Baats, E., et al. (2025). Optical genome mapping enables accurate testing of large repeat expansions. Genome Res. *35*, 810–823. https://doi.org/10.1101/gr.279491.124.

39. Mutlu, M.B., Karakaya, T., Çelebi, H.B.G., Duymuş, F., Seyhan, S., Yılmaz, S., Yiş, U., Atik, T., Yetkin, M.F., and Gümüş, H. (2025). Utility of Optical Genome Mapping in Repeat Disorders. Clin. Genet. *107*, 188–195. https://doi.org/10.1111/cge.14633.

40. Zarouchlioti, C., Efthymiou, S., Facchini, S., Dominik, N., Bhattacharyya, N., Liu, S., Costa, M.A., Szabo, A., Sadan, A.N., Jun, A.S., et al. (2024). Tissue-specific TCF4 triplet repeat instability revealed by optical genome mapping. EBioMedicine *108*, 105328. https://doi.org/10.1016/j.ebiom.2024.105328.

41. van der Sanden, B., Neveling, K., Pang, A.W.C., Shukor, S., Gallagher, M.D., Burke, S.L., Kamsteeg, E.J., Hastie, A., and Hoischen, A. (2024). Optical Genome Mapping for Applications in Repeat Expansion Disorders. Curr. Protoc. *4*, e1094. https://doi.org/10.1002/cpz1.1094.

42. van Agtmaal, E.L., André, L.M., Willemse, M., Cumming, S.A., van Kessel, I.D.G., van den Broek, W.J.A.A., Gourdon, G., Furling, D., Mouly, V., Monckton, D.G., et al. (2017). CRISPR/Cas9-Induced (CTG·CAG)(n) Repeat Instability in the Myotonic Dystrophy Type 1 Locus: Implications for Therapeutic Genome Editing. Mol. Ther. *25*, 24–43. https://doi.org/10.1016/j.ymthe.2016.10.014.

43. Konieczny, P., Stepniak-Konieczna, E., and Sobczak, K. (2014). MBNL proteins and their target RNAs, interaction and splicing regulation. Nucleic Acids Res. *42*, 10873–10887. https://doi.org/10.1093/nar/gku767.

44. Jog, S.P., Paul, S., Dansithong, W., Tring, S., Comai, L., and Reddy, S. (2012). RNA splicing is responsive to MBNL1 dose. PLoS One *7*, e48825. https://doi.org/10.1371/journal.pone.0048825.

45. Bérenger-Currias, N., Martinat, C., and Baghdoyan, S. (2023). Pluripotent Stem Cells in Disease Modeling and Drug Discovery for Myotonic Dystrophy Type 1. Cells *12*. https://doi.org/10.3390/cells12040571.

46. Larsen, J., Pettersson, O.J., Jakobsen, M., Thomsen, R., Pedersen, C.B., Hertz, J.M., Gregersen, N., Corydon, T.J., and Jensen, T.G. (2011). Myoblasts generated by lentiviral mediated MyoD transduction of myotonic dystrophy type 1 (DM1) fibroblasts can be used for assays of therapeutic molecules. BMC Res. Notes *4*, 490. https://doi.org/10.1186/1756-0500-4-490.

47. Nakamori, M., Sobczak, K., Puwanant, A., Welle, S., Eichinger, K., Pandya, S., Dekdebrun, J., Heatwole, C.R., McDermott, M.P., Chen, T., et al. (2013). Splicing biomarkers of disease severity in myotonic dystrophy. Ann. Neurol. *74*, 862–872. https://doi.org/10.1002/ana.23992.

48. Wang, E.T., Treacy, D., Eichinger, K., Struck, A., Estabrook, J., Olafson, H., Wang, T. T., Bhatt, K., Westbrook, T., Sedehizadeh, S., et al. (2019). Transcriptome alterations in myotonic dystrophy skeletal muscle and heart. Hum. Mol. Genet. *28*, 1312–1321. https://doi.org/10.1093/hmg/ddy432.

49. Murillo, A., Alpaugh, M., Larin, M., Randall, E.L., Heraty, L., Durairaj, R.R., Aston, A.N., Taylor, A.S., Monteys, A.M., and Stöberl, N. (2024). Cas9 nickase-mediated contraction of CAG/CTG repeats at multiple disease loci. Preprint at: bioRxiv. https://doi.org/10.1101/2024.02.19.580669

50. Pešović, J., Perić, S., Brkušanin, M., Brajušković, G., Rakočević-Stojanović, V., and Savić-Pavićević, D. (2018). Repeat Interruptions Modify Age at Onset in Myotonic Dystrophy Type 1 by Stabilizing DMPK Expansions in Somatic Cells. Front. Genet. *9*, 601. https://doi.org/10.3389/fgene.2018.00601.

51. Hastie, A.R., Lam, E.T., Pang, A.W.C., Zhang, L.X., Andrews, W., Lee, J., Liang, T.Y., Wang, J., Zhou, X., and Zhu, Z. (2017). Rapid automated large structural variation detection in a diploid genome by nanochannel based next-generation mapping. Preprint at: bioRxiv. https://doi.org/10.1101/102764

52. Neveling, K., Mantere, T., Vermeulen, S., Oorsprong, M., van Beek, R., Kater-Baats, E., Pauper, M., van der Zande, G., Smeets, D., Weghuis, D.O., et al. (2021). Next-generation cytogenetics: Comprehensive assessment of 52 hematological malignancy genomes by optical genome mapping. Am. J. Hum. Genet. *108*, 1423–1435. https://doi.org/10.1016/j.ajhg.2021.06.001.

53. Lam, L.T., Pham, Y.C., Nguyen, T.M., and Morris, G.E. (2000). Characterization of a monoclonal antibody panel shows that the myotonic dystrophy protein kinase, DMPK, is expressed almost exclusively in muscle and heart. Hum. Mol. Genet. *9*, 2167–2173. https://doi.org/10.1093/hmg/9.14.2167.

54. Kaliman, P., and Llagostera, E. (2008). Myotonic dystrophy protein kinase (DMPK) and its role in the pathogenesis of myotonic dystrophy 1. Cell. Signal. *20*, 1935–1941. https://doi.org/10.1016/j.cellsig.2008.05.005.

55. Hale, M.A., Bates, K., Provenzano, M., and Johnson, N.E. (2023). Dynamics and variability of transcriptomic dysregulation in congenital myotonic dystrophy during pediatric development. Hum. Mol. Genet. *32*, 1413–1428. https://doi.org/10.1093/hmg/ddac254.

56. André, L.M., van Cruchten, R.T.P., Willemse, M., Bezstarosti, K., Demmers, J.A.A., van Agtmaal, E.L., Wansink, D.G., and Wieringa, B. (2019). Recovery in the Myogenic Program of Congenital Myotonic Dystrophy Myoblasts after Excision of the Expanded (CTG)n Repeat. Int. J. Mol. Sci. *20*, 5685. https://doi.org/10.3390/ijms20225685.

57. Thornton, C.A., Johnson, K., and Moxley, R.T., 3rd (1994). Myotonic dystrophy patients have larger CTG expansions in skeletal muscle than in leukocytes. Ann. Neurol. *35*, 104–107. https://doi.org/10.1002/ana.410350116.

58. Anvret, M., Ahlberg, G., Grandell, U., Hedberg, B., Johnson, K., and Edström, L. (1993). Larger expansions of the CTG repeat in muscle compared to lymphocytes from patients with myotonic dystrophy. Hum. Mol. Genet. *2*, 1397–1400. https://doi.org/10.1093/hmg/2.9.1397.

59. Nakamori, M., Shimizu, H., Ogawa, K., Hasuike, Y., Nakajima, T., Sakurai, H., Araki, T., Okada, Y., Kakita, A., and Mochizuki, H. (2022). Cell type-specific abnormalities of central nervous system in myotonic dystrophy type 1. Brain Commun. *4*, fcac154. https://doi.org/10.1093/braincomms/fcac154.

60. Gomes-Pereira, M., Cooper, T.A., and Gourdon, G. (2011). Myotonic dystrophy mouse models: towards rational therapy development. Trends Mol. Med. *17*, 506–517. https://doi.org/10.1016/j.molmed.2011.05.004.

61. Gudde, A.E.E.G., González-Barriga, A., van den Broek, W.J.A.A., Wieringa, B., and Wansink, D.G. (2016). A low absolute number of expanded transcripts is involved in myotonic dystrophy type 1 manifestation in muscle. Hum. Mol. Genet. *25*, 1648–1662. https://doi.org/10.1093/hmg/ddw042.

62. Nutter, C.A., Bubenik, J.L., Oliveira, R., Ivankovic, F., Sznajder, Ł.J., Kidd, B.M., Pinto, B.S., Otero, B.A., Carter, H.A., Vitriol, E.A., et al. (2019). Cell-type-specific dysregulation of RNA alternative splicing in short tandem repeat mouse knockin models of myotonic dystrophy. Genes Dev. *33*, 1635–1640. https://doi.org/10.1101/gad.328963.119.

63. Gökirmak, T., Nikan, M., Wiechmann, S., Prakash, T.P., Tanowitz, M., and Seth, P.P. (2021). Overcoming the challenges of tissue delivery for oligonucleotide therapeutics. Trends Pharmacol. Sci. *42*, 588–604. https://doi.org/10.1016/j.tips.2021.04.010.

64. Handal, T., Juster, S., Abu Diab, M., Yanovsky-Dagan, S., Zahdeh, F., Aviel, U., Sarel-Gallily, R., Michael, S., Bnaya, E., Sebban, S., et al. (2024). Differentiation shifts from a reversible to an irreversible heterochromatin state at the DM1 locus. Nat. Commun. *15*, 3270. https://doi.org/10.1038/s41467-024-47217-4.

65. Furling, D., Lemieux, D., Taneja, K., and Puymirat, J. (2001). Decreased levels of myotonic dystrophy protein kinase (DMPK) and delayed differentiation in human myotonic dystrophy myoblasts. Neuromuscul. Disord. *11*, 728–735. https://doi.org/10.1016/s0960-8966(01)00226-7.

66. Rizzo, M., Beffy, P., Del Carratore, R., Falleni, A., Pretini, V., D'Aurizio, R., Botta, A., Evangelista, M., Stoccoro, A., Coppedè, F., et al. (2018). Activation of the interferon type I response rather than autophagy contributes to myogenesis inhibition in congenital DM1 myoblasts. Cell Death Dis. *9*, 1071. https://doi.org/10.1038/s41419-018-1080-1.

67. Beffy, P., Del Carratore, R., Masini, M., Furling, D., Puymirat, J., Masiello, P., and Simili, M. (2010). Altered signal transduction pathways and induction of autophagy in human myotonic dystrophy type 1 myoblasts. Int. J. Biochem. Cell Biol. *42*, 1973–1983. https://doi.org/10.1016/j.biocel.2010.08.010.

68. El Boujnouni, N., van Asbeck, A.H., Dieker, J., Wansink, D.G., and Brock, R. (2022). Imaging of CPP Delivery Mechanisms of Oligonucleotides. Methods Mol. Biol. *2383*, 197–210. https://doi.org/10.1007/978-1-0716-1752-6_13.

69. González-Barriga, A., Mulders, S.A., van de Giessen, J., Hooijer, J.D., Bijl, S., van Kessel, I.D., van Beers, J., van Deutekom, J.C., Fransen, J.A., Wieringa, B., and Wansink, D.G. (2013). Design and analysis of effects of triplet repeat oligonucleotides in cell models for myotonic dystrophy. Mol. Ther. Nucleic Acids *2*, e81. https://doi.org/10.1038/mtna.2013.9.

70. van der Bent, M.L., Wansink, D.G., and Brock, R. (2020). Advanced Fluorescence Imaging to Distinguish Between Intracellular Fractions of Antisense Oligonucleotides. Methods Mol. Biol. *2063*, 119–138. https://doi.org/10.1007/978-1-0716-0138-9_10.

71. Ran, F.A., Hsu, P.D., Wright, J., Agarwala, V., Scott, D.A., and Zhang, F. (2013). Genome engineering using the CRISPR-Cas9 system. Nat. Protoc. *8*, 2281–2308.

72. Mantere, T., Neveling, K., Pebrel-Richard, C., Benoist, M., van der Zande, G., Kater-Baats, E., Baatout, I., van Beek, R., Yammine, T., Oorsprong, M., et al. (2021). Optical genome mapping enables constitutional chromosomal aberration detection. Am. J. Hum. Genet. *108*, 1409–1422. https://doi.org/10.1016/j.ajhg.2021.05.012.

73. Livak, K.J., and Schmittgen, T.D. (2001). Analysis of relative gene expression data using real-time quantitative PCR and the $2^{-\Delta\Delta CT}$ method. Methods *25*, 402–408.

74. Schindelin, J., Arganda-Carreras, I., Frise, E., Kaynig, V., Longair, M., Pietzsch, T., Preibisch, S., Rueden, C., Saalfeld, S., Schmid, B., et al. (2012). Fiji: an open-source platform for biological-image analysis. Nat. Methods *9*, 676–682.

OMTM, Volume *33*

Supplemental information

Repeat length as a key determinant for disease severity and antisense oligonucleotide activity in myotonic dystrophy type 1

Najoua El Boujnouni, Lise Ripken, Marieke Willemse, Bart van der Sanden, Kornelia Neveling, Alexander Hoischen, Roland Brock, and Derick G. Wansink

Supplemental Information

Supplemental Tables and Figures

Table S1: Sequences and modifications of ASOs.[31] Underlined, 2'-O-Me; bold and non-underlined, DNA; *, phosphorothioate (PS) linkage.

Name	Sequence
**Repeat-targeting blocker**	C*A*G*C*A*G*C*A*G*C*A*G*C*A*G
**Control blocker**	G*A*C*G*A*C*G*A*C*G*A*C*G*A*C
***DMPK*-targeting gapmer**	C*G*G*A*G***C*G*G*T*T*G*T*G*A*A***C*U*G*G*C
**Control gapmer**	G*A*C*G***A*C*G*A*C*G*A*C*G*A***C*G*A*C
**Cy5-labelled (repeat-targeting blocker) ASO**	5' Cy5-C*A*G*C*A*G*C*A*G*C*A*G*C*A*G

Table S2: PCR primers used in this study.

Gene	Forward primer (5' → 3')	Reverse primer (5' → 3')	Assay
DMPK e1-2	ACTGGCCCAGGACAAGTACG	CCTCCTTAAGCCTCACCACG	RT-qPCR
DMPK e15 3'	TGCCTGCTTACTCGGGAAATT	GAGCAGCGCAAGTGAGGAG	RT-qPCR
GAPDH	CCCGCTTCGCTCTCTGCTCC	CCTTCCCCATGGTGTCTGAGCG	RT-qPCR
HPRT1	TGACACTGGCAAAACAATGCA	GGTCCTTTTCACCAGCAAGCT	RT-qPCR
MBNL1	AGGGAGATGCTCTCGGGAAAAGTG	GTTGGCTAGAGCCTGTTGGTATTGGAAAATAC	RT-PCR (splicing)
NFIX	GATGGAGAGCCCTGTTGATGACG	GTGGTGGTGGTAGCGGATGGTC	RT-PCR (splicing)
CLASP1	CAAAGTCTCCTCATCTTCGGGCACG	GCTGGGACTGTGAAACCACTTTAGC	RT-PCR (splicing)

Table S3: (CTG)$_n$ repeat length in isogenic Cas9n-treated and control cell lines determined by OGM.

Cell line	Number of triplets
**iDM2900**	2775 (p17)/2955 (p26)
**iDM2200**	2237
**iDM1200**	1204
**iDM3400**	3418
**iDM2700**	2733

Table S4: Common (CNG)$_n$ microsatellites examined in this study.

Gene	Sequence in iDM2900	Forward primer (5' → 3')	Reverse primer (5' → 3')
AR	(CAG)$_{22/23}$ CAA GAG ACT AGC CCC AGG (CAG)$_6$	TAGGGCTGGGAAGGGTCTAC	CTCTGGGACGCAACCTCTCT
ATN1	CAG CAA CAG CAA (CAG)$_8$	ACTCAGCCTTCTCTCCCATC	TGTAGGACACCTGGCTGTGA
ATXN1	(CAG)$_{12}$ CAT CAG CAT (CAG)$_{14/15}$	TTCCAGTTCATTGGGTCCTC	GTGTGTGGGATCATCGTCTG
DMPK	(CTG)$_{13}$	GAAGGGTCCTTGTAGCCGGGAA	GGAGGATGGAACACGGACGG
PPP2R2B	(CAG)$_{10}$	GCAGCAAAGAGCAGCCGCAG	CTGGTCCCACGGGAGGGCGG
TBP	(CAG)$_3$ (CAA)$_3$ (CAG)$_9$ CAA CAG CAA (CAG)$_{16/17}$ CAA CAG	TTCTCCTTGCTTTCCACAGG	GGGGAGGGATACAGTGGAGT
TCF4	(CTG)$_{11}$	CAGCACAAAGCGGAACTTGAC	GGCCATAAACGTGGCAATGTC

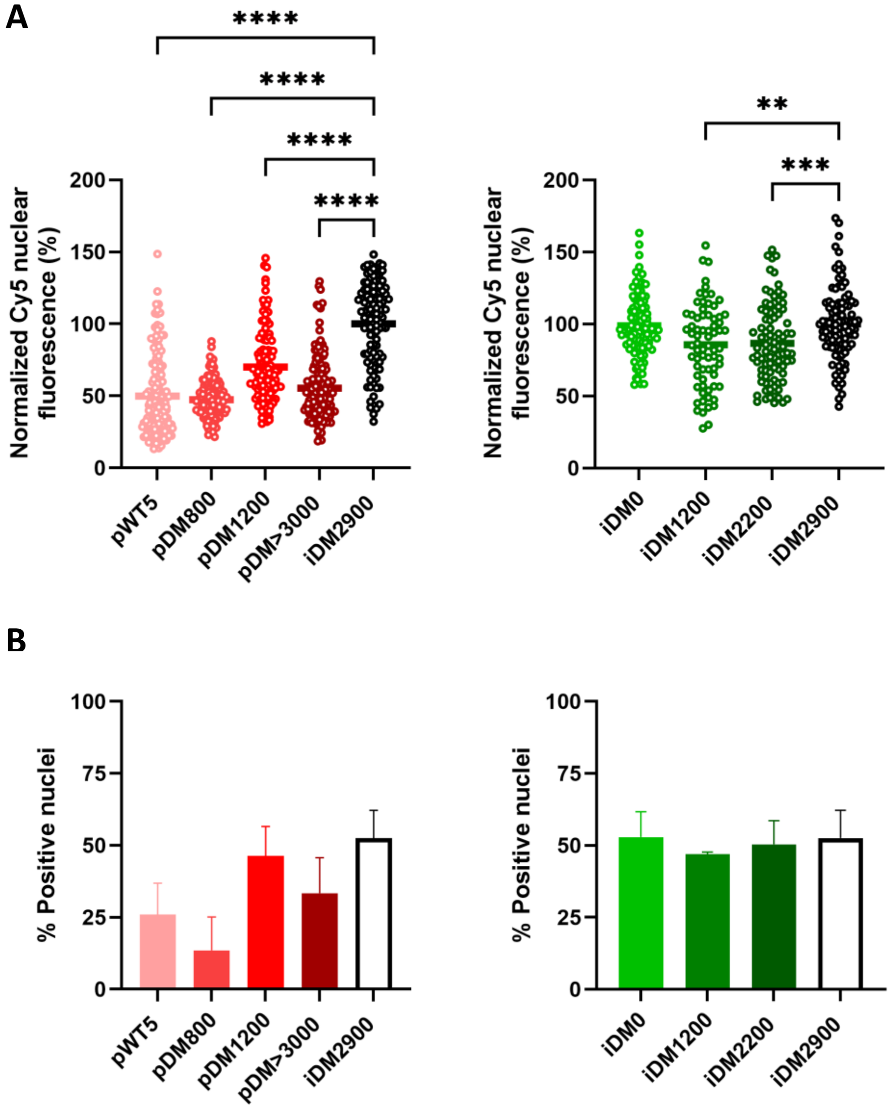

Figure S1: Pepfect14-mediated nuclear uptake of Cy5-labelled ASOs. (A) Mean nuclear (Cy5) fluorescence intensity after treatment with PepFect14/ASO nanoparticles (containing 200 nM Cy5-labelled repeat-targeting blocker ASO) for 24 hours,

together with individual data points (representing imaged nuclei) for the primary (left) and isogenic (right) cell panel. (B) The percentage of transfected ('positive') nuclei for the primary (left) and isogenic (right) cell panel. Data are presented as mean ± SD of a representative experiment (A) or mean ± SEM of three independent experiments (B). A Kruskal-Wallis (nonparametric) test with Dunn post hoc test (A and B (left)) or a one-way ANOVA with Tukey post hoc test (B, right) was used for comparison of nuclear uptake between all cell cultures. **$p < 0.01$. ***$p < 0.001$, ****$p < 0.0001$.
